# A Technology-Assisted Telephone Intervention for Work-Related Stress Management: Pilot Randomized Controlled Trial

**DOI:** 10.2196/26569

**Published:** 2022-07-13

**Authors:** Salla Tuulikki Muuraiskangas, Anita Marianne Honka, Ulla-Maija Junno, Hannu Olavi Nieminen, Jouni Kalevi Kaartinen

**Affiliations:** 1 Smart Health VTT Technical Research Centre of Finland Ltd Oulu Finland; 2 Smart Health VTT Technical Research Centre of Finland Ltd Tampere Finland; 3 Luona Hoiva Ltd Helsinki Finland; 4 Movendos Ltd Tampere Finland; 5 Faculty of Medicine and Health Technology Tampere university Tampere Finland

**Keywords:** health behavior change intervention, telephone coaching, technology-assisted coaching, remote coaching, occupational health, mental well-being, stress management, feasibility, randomized controlled trial

## Abstract

**Background:**

Stress management interventions combining technology with human involvement have the potential to improve the cost-effectiveness of solely human-delivered interventions, but few randomized controlled trials exist for assessing the cost-effectiveness of technology-assisted human interventions.

**Objective:**

The aim of this study was to investigate whether a technology-assisted telephone intervention for stress management is feasible for increasing mental well-being or decreasing the time use of coaches (as an approximation of intervention cost) while maintaining participants’ adherence and satisfaction compared with traditional telephone coaching.

**Methods:**

A 2-arm, pilot randomized controlled trial of 9 months for stress management (4-month intensive and 5-month maintenance phases) was conducted. Participants were recruited on the web through a regional occupational health care provider and randomized equally to a research (technology-assisted telephone intervention) and a control (traditional telephone intervention) group. The coaching methodology was based on habit formation, motivational interviewing, and the transtheoretical model. For the research group, technology supported both coaches and participants in identifying behavior change targets, setting the initial coaching plan, monitoring progress, and communication. The pilot outcome was intervention feasibility, measured primarily by self-assessed mental well-being (WorkOptimum index) and self-reported time use of coaches and secondarily by participants’ adherence and satisfaction.

**Results:**

A total of 49 eligible participants were randomized to the research (n=24) and control (n=25) groups. Most participants were middle-aged (mean 46.26, SD 9.74 years) and female (47/49, 96%). Mental well-being improved significantly in both groups (WorkOptimum from “at risk” to “good” Â>0.85; *P*<.001), and no between-group differences were observed in the end (Â=0.56, 95% CI 0.37-0.74; *P*=.56). The total time use of coaches did not differ significantly between the groups (366.0 vs 343.0 minutes, Â=0.60, 95% CI 0.33-0.85; *P*=.48). Regarding adherence, the dropout rate was 13% (3/24) and 24% (6/25), and the mean adherence rate to coaching calls was 92% and 86% for the research and control groups, respectively; the frequency of performing coaching tasks was similar for both groups after both phases; and the diligence in performing the tasks during the intensive phase was better for the research group (5.0 vs 4.0, Â=0.58, 95% CI 0.51-0.65; *P*=.03), but no difference was observed during the maintenance phase. Satisfaction was higher in the research group during the intensive phase (5.0 vs 4.0, Â=0.66, 95% CI 0.58-0.73; *P*<.001) but not during the maintenance phase.

**Conclusions:**

The technology-assisted telephone intervention is feasible with some modifications, as it had similar preliminary effectiveness as the traditional telephone intervention, and the participants had better satisfaction with and similar or better adherence to the intervention, but it did not reduce the time use of coaches. The technology should be improved to provide more digested information for action planning and templates for messaging.

**Trial Registration:**

ClinicalTrials.gov NCT02445950; https://www.clinicaltrials.gov/ct2/show/study/NCT02445950

## Introduction

### Background

Work-related stress and its indirect consequences for physical, mental, and social well-being are serious threats to public health. Long-term stress increases the risk of sleep problems [1,2], coronary heart disease [3,4], and various mental health problems [5], such as burnout [6,7], anxiety [8], and depression [9,10]. Stress is also linked to an increased number of sick days [11-15], lost productivity, inability to work, and presenteeism at work [16-18]. The estimated stress-related health, economic, and social costs are considerable [13,14,19]. Hence, reducing work-related stress effectively can have a major positive impact on individuals, companies, and society as a whole.

Various interventions have been developed for stress management and mental well-being. Traditionally, interventions have been delivered by human coaches or therapists through face-to-face or telephone sessions, but such interventions are not easily scalable. Nowadays, interventions are often supported by technology, or they can even be delivered fully and automatically by technology without human involvement. The use of technology can decrease human involvement and thus costs [20], which enables scaling up the intervention for a larger population, and therefore, it holds a promise for a healthier population.

Stress management interventions that blend technology and human effort have been found in several studies to be effective at reducing stress compared with no treatment [21-23]. In addition, there is evidence from the mental health perspective that blended interventions are more effective at reducing symptoms of anxiety, distress, and negative thoughts than, for example, fully human interventions [24,25]. Fully automatized interventions without human involvement have also been found to be effective in reducing stress [23,26-30]. However, there are studies indicating that blended stress management interventions can be more effective at reducing stress or stress-related mental health problems than fully technological interventions [31,32].

The level of human and technology involvement and the terminology used to describe the blend varies in health intervention studies [20,33]. In this study, we focus on *technology-assisted human interventions*, where the intervention is produced by a human (therapist, coach, etc) with the assistance of technology. Technology assists the human by facilitating the coaching process or by providing additional insights regarding the participant via automatic analyses of electronic questionnaire responses or passively collected data (eg, via wearables). An example of this kind of intervention is telephone coaching, where technology provides additional insights (eg, participants’ self-monitoring data or intervention component suggestions) to support the decision-making of coaches [23,34].

There are only a few randomized controlled trials (RCTs) studying the cost-effectiveness of blended stress management interventions. These studies have shown blended stress management interventions to have an acceptable likelihood for cost-effectiveness compared with waiting list control [35-37], fully human interventions with no technology involved, [38] and fully technological interventions [39]. In addition, blended interventions for mental health are cost-effective compared with fully human interventions, at least from the care provider perspective if not from the societal perspective [40]. In these studies, cost-effectiveness was calculated by comparing medical and societal costs to the measured health improvements [36-38,40] or to the number of patients with a symptom-free status [35]. For instance, cost-effectiveness was evaluated based on the total therapist time spent compared with the measured health improvements in the study by Kaldo et al [38].

Participants’ intervention adherence is an important determinant of intervention effectiveness [41] and feasibility, as it defines participants’ exposure to the intervention. It is necessary to distinguish between at least two types of adherence, the adherence to the research study and its questionnaires (conversely called dropout attrition) and adherence to the intervention itself, that is, to what extent the components of the intervention are being used (conversely called nonuse attrition) [42]. Previous studies on mental health suggest that intervention adherence (both regarding the study and the use) is higher when human guidance is involved in the intervention [20,41,43-45].

Participants’ satisfaction with the intervention is important for intervention feasibility, and it is associated with sustained adherence [46]. Therefore, studying satisfaction sheds light on understanding intervention adherence and effectiveness. The scientific publications studying participant satisfaction in blended stress management interventions are scarce. Earlier studies on mental health [47] have shown that in interventions in which the technology is used more and human assistance is less, the participants are less satisfied than in fully human interventions [47], but there is also evidence of equal satisfaction among the participants in blended interventions and group-based, fully human interventions [48].

In summary, previous research suggests that blended stress management interventions have the potential to be effective, but cost-effectiveness studies are lacking. Furthermore, adherence and satisfaction are important for evaluating the feasibility of interventions in more detail and for helping to refine their implementation for future large-scale RCTs.

### Objectives

The primary objective of this study was to investigate whether a technology-assisted telephone intervention for stress management is feasible for increasing participants’ well-being or decreasing the time use of coaches while maintaining participants’ adherence and satisfaction compared with a traditional telephone intervention (without technology assistance) in an occupational health care setting. The primary trial outcomes were mental well-being and time use of coaches as an approximation of the intervention cost (ClinicalTrials.gov NCT02445950). As primary analyses, we assessed whether the participants in the research group (technology-assisted telephone intervention) reported a greater improvement in well-being, measured by the WorkOptimum index [49,50], and whether the time use of coaches was lower than that of the control group (telephone intervention). As secondary outcomes, we assessed participants’ adherence and satisfaction in both groups. The secondary outcomes mentioned in the trial registration related to the evaluation of the used technology (usefulness, ease of use, and accuracy) will be reported in another paper (Honka, MSc, unpublished data, December 2021).

## Methods

### Trial Design

A nonblinded, parallel-group, 2-arm pilot RCT was conducted for 9 months in Oulu, Finland, to explore whether a technology-assisted telephone intervention for stress management is feasible for increasing mental well-being or decreasing the time use of coaches while maintaining adherence and satisfaction. The trial registration opened in November 2014, and the trial started in February 2015 and ended in October 2015.

### Participants

Participants were recruited from among the employees of the City of Oulu, Finland, via the channels of the regional occupational health care provider. The recruitment announcement was published on the intranet pages of the City of Oulu and the occupational health care provider, and in the magazines of the City of Oulu (to staff) and the occupational health care provider (to customers). The staff of the occupational health care provider also recruited participants personally and via email. The registration of the study was conducted on the web via a link in the announcement. Registered employees received informed consent through regular mail, where information regarding the 2 study groups was provided: intervention, data collection, data processing, data privacy, research partners, and contact details. Signed consent was collected by a research partner who provided the coaching service for the intervention. An electronic eligibility survey was sent to the employees who returned signed consent forms, after which they were informed whether they were accepted to the study or not. The eligibility criteria are presented in Textbox 1. As the quality of romantic relationships substantially influences mental well-being [51], this was selected as one of the intervention areas; therefore, being in a relationship was part of the inclusion criteria.

**Eligibility criteria for the study.**

**Inclusion criteria**

- Own assessment of decreased psychophysical state (based on a subset of items of the WorkOptimum questionnaire)
- Customers of the occupational health care provider who work full-time for the City of Oulu (in the area of information technology, education, culture, social, health, and customer service)
- Age >18 years
- In a relationship, motivated to enhance own well-being by making lifestyle changes or performing exercises related to mental well-being or relationships (based on 1 question in the eligibility survey)

**Exclusion criteria**

- Night shifts included in the work schedule
- Acute health condition or a serious disease
- Chronic pain affecting physical function
- Long period of absence (eg, long vacation, alternation leave, parental leave, or pension) from work during the intervention period
- Participation in other studies

### Randomization

The eligible participants were randomly allocated to either a research (technology-assisted telephone intervention) or control (traditional telephone intervention) group in a 1:1 ratio through stratified block randomization. Group allocation was stratified based on socioeconomic status and having minors as family members, since these factors were anticipated to influence the mental well-being and adherence outcomes of the study owing to challenges in meeting the demands of work and family responsibilities [52-54]. Socioeconomic status was categorized as “lower-level employees” (eg, secretaries, nurses, childminders, and customer servants) or “upper-level employees” (eg, managers, doctors, psychologists, teachers, and information technology professionals) based on job descriptions and the required education level [55]. Having minors in the family was described using 3 categories: “no children,” “at least one child below school age (<7 years),” and “only school-aged children.”

The participants were randomized simultaneously into the 2 groups via Microsoft Excel (version 2010) using its random number generator. The randomization was conducted by a researcher who was not involved in the study as an investigator. The study investigators were aware of the group to which each participant belonged.

### Sample Size

Available coaching resources defined the number of participants that could be enrolled in the study. At the time of the study, the participating coaching service provider used 3 coaches who could use an average of 20% of their time for the study participants. Therefore, the objective was to have 40 participants in the study. As a dropout rate of 20% is common in telephone interventions [34,56], the aim was to recruit 50 participants.

### Interventions

#### Common Intervention Components

**Figure 1:**
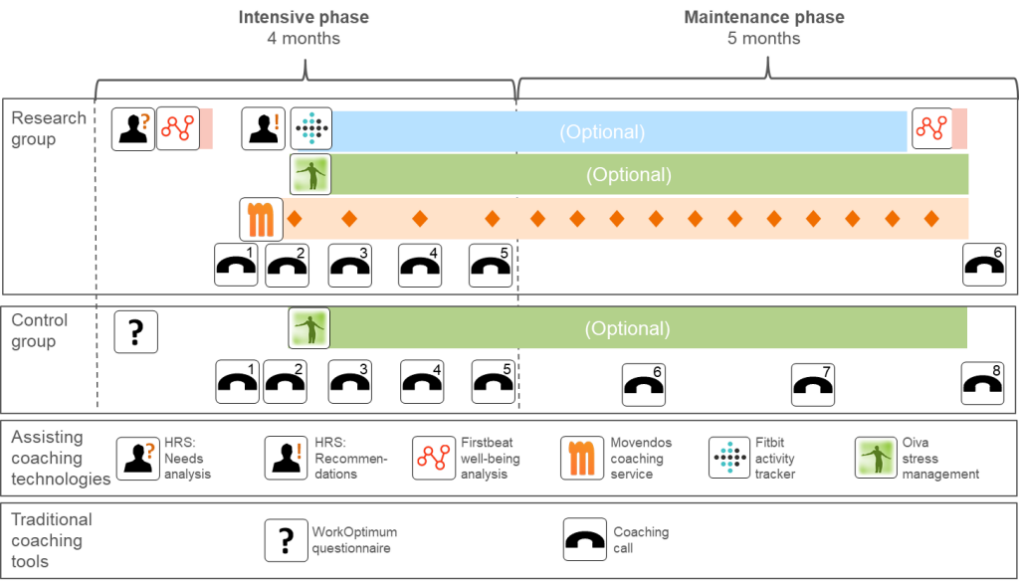
Intervention timeline with intervention components. HRS: health recommender system.

There were 2 interventions: a technology-assisted telephone intervention and a traditional telephone intervention. The interventions lasted for 9 months, and they were divided into a 4-month intensive phase and a 5-month maintenance phase (Figure 1).

Coaching was performed by 3 coaches recruited by the research partner, Mawell Care Limited, so that each of them had an equal number of clients from both the control and research groups. The participants were allocated to the coaches based on mutual availability for the first telephone call. Coaching is based on the habit formation theory, according to which small, regularly repeating behavioral actions or tasks support long-term behavior change [57]. The tasks were related to different topics relevant to well-being, namely sleep, physical activity, eating, alcohol consumption, smoking, recovery from stress, anxiety, personal values, workload management, quality of relationship, self-esteem, and weight management. The tasks could be, for example, “take at least 7000 steps a day,” “practice relaxation with the help of audio exercises,” and “keep a diary of personal eating habits for some days.” Some of these tasks included links to external web resources, such as “read information about the consequences of sleep deprivation from [webpage],” and “practice mindful eating with Oiva at a weekly basis.” Oiva is a web portal that includes exercises for mental well-being, which are based on Acceptance and Commitment Therapy [58]. Oiva contains short few-minute exercises under the themes of well-being of mind and body, values, and everyday choices. The number of study participants per study group, working on different task areas, is presented in Table 1.

Behavioral strategies were based on motivational interviewing and the transtheoretical model of Prochaska et al [59,60]. The interventions included several behavior change techniques, as categorized in the study by Michie et al [61]. The applied behavior change techniques are presented in Table 2.

The main difference between the interventions was the number of telephone calls and the use of technology in coaching. The research group had 5 coaching calls during the intensive phase and 1 at the end. The control group had 5 coaching calls in the intensive phase and 3 in the maintenance phase.

**Table 1:** Task areas and selection frequency for both groups.

Task areas	Group, n^a^ (%)		Total, n (%)
	Research	Control	
Sleep	7 (58)	5 (42)	12 (100)
Physical activity	23 (55)	19 (45)	42 (100)
Eating	9 (47)	10 (53)	19 (100)
Alcohol consumption	1 (50)	1 (50)	2 (100)
Smoking	1 (50)	1 (50)	2 (100)
Recovery from stress, anxiety, or personal values	20 (63)	12 (37)	32 (100)
Workload management	9 (53)	8 (47)	17 (100)
Quality of relationship	3 (100)	0 (0)	3 (100)
Self-esteem	3 (100)	0 (0)	3 (100)
Weight management	5 (45)	6 (55)	11 (100)

^a^Number of participants in a group who selected a task from a specific area.

**Table 2:** Behavior change techniques used in both interventions.

Phase of the intervention	Behavior change technique
Beginning	Goal setting of behavior Goal setting of outcome Problem solving Action planning Behavioral contact Information about health consequences Pros and cons Comparative imagining of future outcomes
Coaching during intensive and maintenance phases	Reviewing behavior goals Feedback on behavior Self-monitoring of behavior Social support (unspecified) Instructions on how to perform the behavior Habit formation Credible source Social reward Reduce negative emotions Verbal persuasion about capability
Final call	Reviewing outcome goals

#### Technology-Assisted Telephone Intervention

##### Overview

In the research group, technology was used for supporting both the coach and participants throughout the intervention. Web tools and wearables were used to support the identification of the participants’ behavior change targets, the creation of the initial intervention plan, progress monitoring, and communication. Technology was designed to help coaches obtain an accurate and comprehensive picture of the participants’ situation (needs and motivation) efficiently in a systematic manner to enhance coaching quality and reduce the time needed to acquire this in-depth knowledge, as well as to empower the participants to be more active in planning and performing their behavior change actions. These technological tools were new to coaches. Before using the tools, the coaches were offered training during four 6-hour information sessions, where the study protocol and other practical issues were also presented. The technology used was free for the participants, and they were encouraged to use it in their everyday lives.

At the beginning of the intensive phase, the research group received Firstbeat heart rate variability (HRV) sensors and wore them for 3 days (Firstbeat Technologies Ltd, see more details below in Firstbeat Well-being Analysis section) [62]. The coach prepared for the first coaching call by reviewing the results of the Firstbeat well-being analysis and the health recommender system’s (HRS’s) behavior change needs analysis (see more details below in HRS: Behavior Change Needs Analysis and Coaching Task Recommendation section). The first call was about discussing the change needs (based on Firstbeat well-being analysis and the HRS’s behavior change need analysis), agreeing with a high-level behavior change goal (eg, sleep better and manage workload), and guiding the participant to select up to 3 coaching tasks via the HRS before the next call. The participants could select tasks either from a recommended list of items or from a task library that includes all the task items available or create their own tasks. After each call, the coaches made notes related to the call (what was agreed and how long the call lasted). Before the second call, the coach reviewed the preselected tasks. During the second call, the preselected tasks were adjusted and the coaching plan was finalized (selecting goals and tasks). If physical activity tasks were selected and the participant wanted a wearable, a Fitbit activity bracelet (Fitbit Inc) [63] was provided. The selected tasks were transferred automatically to the Movendos coaching web service (more details below in Movendos Coaching Service section) [64,65]. For stress management tasks, the Oiva stress management web service was offered to the participants [58]. Coaching calls 2 to 5 were used to discuss the suitability of the goals, supporting the change through motivational interviewing, and updating the coaching plan. Before each call, the coach reviewed the progress from Movendos regarding performing the agreed coaching tasks.

During the maintenance phase, the research group received coaching only via Movendos messages. The coaches were expected to send group messages to the research group once a month and personal coaching messages every 2 weeks in addition to replying to any messages from the participants weekly. Before sending the messages, the coaches checked the progress of the participants on Movendos. The coaching messages then focused on motivating them to perform tasks that did not progress. The research group repeated the Firstbeat well-being analysis at the end of the maintenance phase, and the appropriate timings for the Firstbeat measurements and the final coaching call were agreed upon over a phone conversation between the coach and participant. The sixth and the last coaching call was used for going through the results (Firstbeat well-being analysis), the coaching experience, and forming a plan for the time after coaching. In the following section, we describe each of the technologies used in greater detail.

##### Firstbeat Well-being Analysis

Firstbeat well-being analysis (Firstbeat Technologies Ltd) [62] provides an analysis of the balance between stress and recovery based on HRV. The participants wore the electrodes for 3 days, and based on the HRV data, their physiological well-being was assessed.

##### HRS: Behavior Change Needs Analysis and Coaching Task Recommendation

During the project, a web-based HRS was developed to analyze participants’ behavior change need areas and to provide personalized recommendations for suitable behavior change actions, that is, coaching tasks, based on the identified needs. The HRS evaluated several well-being–related or lifestyle-related areas (sleep sufficiency and quality, eating rhythm, balanced diet, emotional eating, physical activity, alcohol consumption, smoking, workload management, recovery from stress, anxiety, personal values, quality of relationship, and self-esteem) based on questionnaires and the results of the Firstbeat well-being analysis. As a result, the HRS provided a report summarizing for each of these areas the strength of the behavior change need (on a scale of 1 to 5) and the readiness to change the behavior categorized by the transtheoretical model’s stages of change [66]. On the basis of the behavior change needs analysis, the HRS provided a list of coaching tasks recommended for inclusion in the coaching plan. In addition, the complete list of available coaching tasks can be explored via the HRS, which includes >100 tasks regarding different areas of well-being and health behaviors. Both the coach and participants could access the information provided by the HRS.

##### Movendos Coaching Service

The research group used the Movendos coaching web service (version 1.27; Movendos Ltd) [64,65] for (1) communicating with the coach via messages (eg, feedback), (2) progress monitoring, and (3) receiving reminders from the coach and setting reminders for themselves if they wished. The coaches had access to a wide library of tasks in the Movendos coaching service, which they could assign to participants, and the service provided them information on the participants’ progress regarding the selected tasks.

##### Telephone Intervention

The control group received 8 coaching calls in total: 5 in the intensive phase and 3 in the maintenance phase. Before the first coaching call, the coaches reviewed the WorkOptimum questionnaire results (administered as a part of the eligibility questionnaire) [49,50]. The first call was about discussing change needs (based on the WorkOptimum index) and forming the coaching plan (selecting goals and tasks). After each call, the coaches made notes related to the call (what was agreed and how long the call lasted). Calls 2 to 7 were used for discussing the suitability of the tasks, supporting the change through motivational interviewing, and updating the coaching plan. The eighth and final call was used for going through the repeated WorkOptimum questionnaire results, the coaching experience, and forming a plan for the time after coaching.

### Outcome Measures

#### Overview

The pilot outcome was intervention feasibility measured primarily by the participants’ self-assessed mental well-being and the total time use of the coaches for the complete coaching period and secondarily by participants’ adherence to and satisfaction with coaching.

The feasibility criteria are formulated in the following manner:

- *Stop—main study not feasible* if the research group has lower well-being than the control group.
- *Continue, but modify intervention—feasible with modifications* if the research group has in comparison with the control group (1) similar well-being and similar or increased time use of coaches, (2) similar well-being and decreased time use of coaches but poorer adherence or satisfaction, or (3) improved well-being and increased time use of coaches.
- *Continue without modifications—feasible but monitor closely* if the research group has in comparison with the control group (1) improved well-being and similar or decreased time use of coaches or (2) similar well-being, decreased time use of coaches, and similar or better adherence or satisfaction.
- *Continue without modifications—feasible as is* if the research group has improved well-being and decreased time use of coaches in combination with similar or better adherence and satisfaction.

#### Mental Well-being

Mental well-being was assessed using the WorkOptimum index, which is a measure of occupational health, and aims to detect work-related cognitive decline and decrease in mental well-being before developing mental health problems (Multimedia Appendix 1 [49,50]). The scoring of the index is divided into 4 categories with the following interpretations: exhaustion (score −4 or less), high-risk (score −3.9 to −2.5), at risk (score −2.4 to −1.0), and good (score −0.9 to 0.0). The WorkOptimum questionnaire evaluates the ability to recover from work and perceived mental and physical resources as well as the perceived energy level and workload. The electronic questionnaire was administered to the participants at baseline (month 0) and at the end of the intensive (month 4) and maintenance (month 9) phases.

#### Time Use of Coaches

The total time use of coaches was tracked during the entire intervention regarding (1) *preparation time* for the coaching calls, (2) *duration* of the coaching calls (ie, 6 calls for the research group and 8 calls for the control group), and (3) the time spent on *writing personal coaching messages* to the research group. For each coaching call, the coaches were asked to manually record the duration of the call and the time spent preparing for it (in minutes). On the basis of these results, the total time spent on coaching calls per participant was computed. The time spent on messaging had to be estimated, as the coaches considered it too laborious to record the time used for messages. The estimate was computed based on the total number of messages sent to each of the research group participants (recorded by the Movendos system log) and the results of previous studies. For the intensive phase, 5 minutes was considered as the time estimate for responding to a participant's message, which is comparable with the time it took physicians to address emails in the study by Leong et al [67]. For the maintenance phase, 10 minutes were assigned as the time estimate per message [68], as coaches were required to initiate motivating coaching messages in addition to just responding to participants. The total time used by coaches was computed by adding the total time spent on coaching calls and messages together, per participant. In addition, the mean preparation time and mean duration per coaching call were computed for each participant.

Only participants for whom all the planned coaching calls were realized and the time-keeping records were complete for both call-preparation time and call duration were included in the analysis of the total time use of coaches. For 14 participants (7 participants from both groups), the time records for the coaching calls were incomplete because one of the coaches recorded only the time spent on preparation activities but missed recording the duration of the calls. Hence, complete data were available only for 11 participants per group.

#### Adherence

Adherence was assessed by the dropout attrition, describing how many participants quit the intervention, and by the use adherence (inversely nonuse attrition). The use adherence comprised (1) the proportion of realized coaching calls, (2) the frequency of performing the selected coaching tasks, and (3) diligence in performing the tasks. During the intensive phase, the coaches evaluated the task performance adherence (frequency and diligence) 3 times for the research group (during coaching calls 3-5) and 4 times for the control group (during calls 2-5) via a structured interview (Multimedia Appendix 2). For each coaching task, the following 3 items were assessed: “the client performed the task less frequently than agreed,” “the client performed the task more frequently than agreed,” and “the client performed the task with diligence,” with a 5-point Likert scale (1=“strongly disagree”; 5=“strongly agree”) also having the option “I don’t know.” For the statistical analyses, the answers to the items related to task performance frequency (2 items) were combined and transformed to a scale from 1 to 9 with the following meaning: 1=“the task was performed less frequently than agreed,” 5=“the task was performed as agreed,” and 9=“the task was performed more frequently than agreed.” During the maintenance phase, the participants self-assessed their task performance adherence for each coaching task via 3 electronic questionnaires administered at months 5, 7, and 9 (after calls 6-8 for the control group; Multimedia Appendix 2). The task performance frequency was assessed with the item “how actively did you perform the coaching task?” with a 5-point Likert scale (1=“less frequently than agreed,” 3=“as agreed,” 5=“more frequently than agreed”). The task performance diligence was assessed with the item “I performed the task with diligence” with a 5-point Likert scale (1=“strongly disagree”; 5=“strongly agree”). Both items included the response option “I don’t know.” For each assessment point, the evaluations for the 2 best-evaluated tasks were considered in the analyses, as the number of selected tasks varied per person and per coaching phase. Group-level medians were calculated for the intensive and maintenance phases over all the tasks considered.

#### Satisfaction With Coaching

Participants’ satisfaction with coaching was assessed using 1 question in different phases of the trial. For the research group, during the intensive phase, the statement was “I was satisfied with the coaching call,” and during the maintenance phase, the statement was “I was satisfied with the coaching received via Movendos messages.” For the control group, the statement remained the same throughout the intervention, that is, “I was satisfied with the coaching call.” The 5-point Likert scale was used (1 = “Strongly disagree”; 5 = “Strongly agree”). Group-level medians were calculated over all the assessments available for each group for the intensive and maintenance phases, as well as for the entire intervention. Satisfaction was assessed at 4 time points (after calls 2-5 for the research group, and after calls 3-5 for the control group) during the intensive phase and 4 time points in the maintenance phase (after calls 6-8 for the control group).

### Statistical Methods

For the primary trial outcome (WorkOptimum index for mental well-being), Mann-Whitney *U* tests were conducted on the follow-up scores measured at the end of the intensive and maintenance phases to determine the statistical significance of the between-group differences. Analysis of covariance was considered for the statistical tests, but as the residuals were not normally distributed, it was more appropriate to use nonparametric tests instead of parametric ones. Similar between-group analyses were performed for the time use of coaches and participants’ adherence to (frequency and diligence) and satisfaction with coaching. In addition, the statistical significance of the within-group changes in mental well-being from baseline (month 0) to the end of the intensive (month 4) and maintenance phases (month 9) were determined using the Sign test. For both groups, the number of participants with positive changes (N_+_) was reported as a test statistic for the Sign test. For the different outcomes, all participants with relevant data available were included in the analyses (available-case analysis). A significance level (α) of .05 was used for the statistical tests. Statistical tests were performed using IBM SPSS Statistics (version 25).

The Vargha-Delaney A measure of stochastic superiority [69] was reported as an indicator of the effect size. For the between-group analyses, 95% CIs of the effect sizes were reported. The effect size computations were performed with free R (version 4.0.5) statistical software by using the *rcompanion* package. The 95% CIs were computed using the bootstrap procedure [70].

### Ethics Approval

This study was approved by the Ethics Committee of Human Sciences at the University of Oulu. The RCT was registered at ClinicalTrials.gov (NCT02445950). Informed consent was obtained from interested individuals through regular mail before administering the electronic eligibility survey via email.

## Results

### Participants

**Figure 2:**
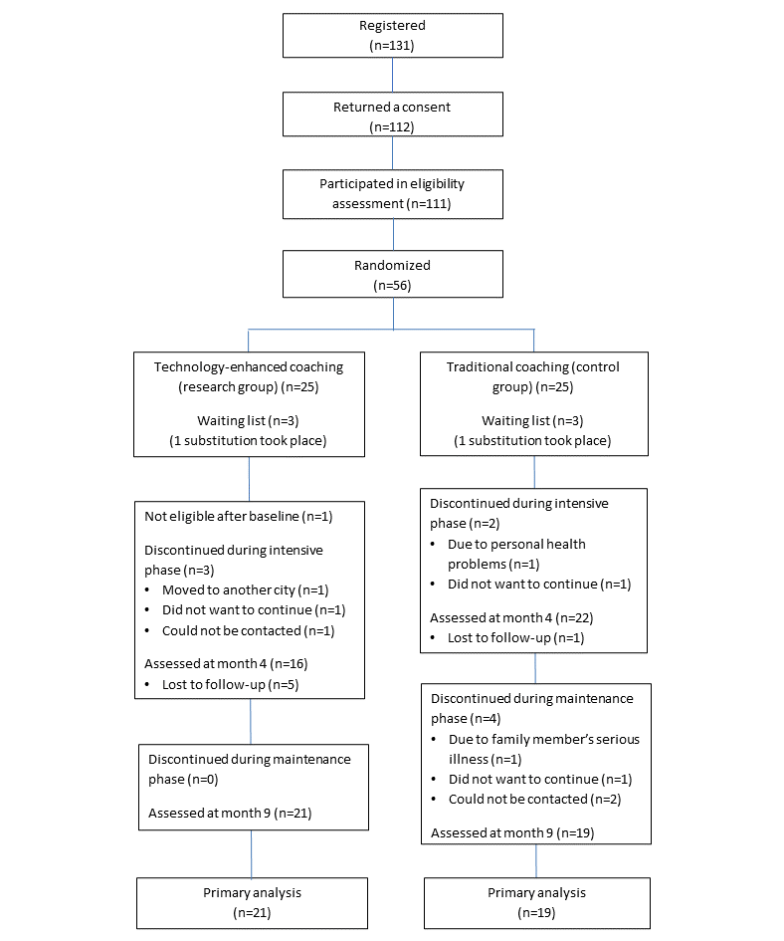
Participant flow for the primary analysis regarding mental well-being.

In total, 131 volunteers registered for the study, of which 56 (42.7%) met the inclusion criteria and were randomized equally to the research and control groups. Of the 56 randomized group of participants, 50 (89%) were chosen to be enrolled in the study based on the order of registration. The remaining 6 participants were put on a waiting list in case of last-minute changes in participation before starting the coaching program. At the beginning of the coaching program, 1 participant in the research group was no longer eligible for the study because of a change in their employment status and was therefore omitted from the statistical analyses. Figure 2 summarizes the participant flow from registration to available-case analysis for mental well-being (primary outcome) and the attrition numbers for the intensive and maintenance phases together with the reasons for withdrawal.

The baseline characteristics of the study participants are presented in Table 3. Most participants (47/49, 96%) were female, and the 2 (4%) male participants were allocated to the control group. More than half (28/49, 57%) of the participants were aged between 46 and 60 years (mean age 46.26, SD 9.74 years). A majority had at least a bachelor’s degree (41/49, 84%). More than half (28/49, 57%) of the participants had a lower socioeconomic status, and approximately half (25/49, 51%) of the participants had children (school-aged or younger). The 2 groups had similar characteristics apart from education level, which was higher in the control group (4/24, 17%, vs 10/25, 40% having a graduate or doctoral degree). Mental well-being at the baseline was at the “at risk” level for both groups (−2.38 vs −2.14) in terms of the interpretation of the median WorkOptimum index (primary outcome).

**Table 3:** Baseline characteristics.

		Research (n=24), n (%)	Control (n=25), n (%)	All (n=49), n (%)
**Gender**				
	Female	24 (100)	23 (92)	47 (96)
**Age (years)**				
	26-35	5 (21)	4 (16)	9 (18)
	36-45	6 (25)	6 (24)	12 (24)
	46-60	13 (54)	15 (60)	28 (57)
**Education**				
	Secondary school	5 (21)	3 (12)	8 (16)
	Bachelor’s degree	15 (63)	12 (48)	27 (55)
	Graduate or doctoral degree	4 (17)	10 (40)	14 (29)
**Socioeconomic status^a^**				
	Lower-level employees	13 (54)	15 (60)	28 (57)
	Upper-level employees	11 (46)	10 (40)	21 (43)
**Family**				
	No children	13 (54)	11 (44)	24 (49)
	At least 1 child below school age (<7 years)	4 (17)	5 (20)	9 (18)
	Only school-aged children	7 (29)	9 (36)	16 (33)

^a^Socioeconomic groups are based on the classification of Statistics Finland [55]. Lower-level employees include, for example, secretaries, nurses, childminders, customer service staff, and assistants. Upper-level employees include, for example, managers, doctors, psychologists, teachers, and information technology professionals.

### Mental Well-being

The follow-up scores for the mental well-being measure, WorkOptimum index (primary outcome), at the end of intensive (month 4: −0.95 vs −1.09, Â=0.53, 95% CI 0.34-0.72; *P*=.74) and maintenance (month 9: −0.47 vs −0.44, Â=0.56, 95% CI 0.37-0.74; *P*=.56) phases were not statistically significantly different between the research and control groups (Table 4). At month 9, the WorkOptimum index had improved from the baseline’s “at risk” level (−2.4 to −1.0) to a “good” (−0.9 to 0.0) level in both groups. The within-group improvements were strong for both groups (Â>0.85, *P*≤.001 at month 9; Table 5). The distribution of WorkOptimum value categories across time points is presented in Table 6.

**Table 4:** Between-group differences of the outcome measures.

Outcome			Research		Control		Mann-Whitney *U* statistic	*P* value	Â^a^ (95% CI)
			Median (25th; 75th) or % (range)	n	Median (25th; 75th) or % (range)	n			
**Mental well-being^b^**									
	Month 0		−2.38 (−3.09; −1.19)	24	−2.14 (−5.50; −1.28)	25	N/A^c^	N/A	N/A
	Month 4		−0.95 (−1.33; −0.50)	16	−1.09 (−1.80; −0.46)	22	188.00	.74	0.53 (0.34-0.72)
	Month 9		−0.47 (−0.72; −0.15)	21	−0.44 (−1.73; −0.26)	19	221.50	.56	0.56 (0.37-0.74)
**Time use of the coaches (minutes)**									
	**Total time use**								
		Months 0-9	366.0 (320.0; 427.0)	11	343.0 (268.0; 489.0)	11	49.0	.48	0.60 (0.33-0.85)
	**Mean preparation time per call**								
		Months 0-9	30.00 (24.0; 32.60)	11	17.57 (16.14; 23.0)	11	12.50	.001	0.90 (0.75-1.0)
	**Mean duration per call**								
		Months 0-9	25.50 (20.33; 30.50)	17	22.44 (17.28; 32.44)	14	97.0	.40	0.59 (0.37-0.80)
**Adherence**									
	**Dropout attrition (%)**								
		Months 0-4	12.5^d^	24	8^d^	25	N/A	N/A	N/A
		Months 4-9	0^d^	24	16^d^	25	N/A	N/A	N/A
		Months 0-9	13^d^	24	24^d^	25	N/A	N/A	N/A
	**Mean adherence to coaching calls (%)**								
		Month 9	92 (50.0-100.0)	24	86 (25.0-100.0)	25	N/A	N/A	N/A
	**Frequency of performing the tasks**								
		Month 4 (scale 1 to 9)	5.0 (2.0; 5.0)	23	5.0 (2.0; 5.0)	24	6543.50	.95	0.50 (0.42-0.57)
		Month 9 (scale 1 to 5)	3.0 (2.0; 3.0)	20	3.0 (2.0; 3.0)	16	2126.0	.37	0.54 (0.45-0.62)
	**Diligence in performing the tasks (scale 1 to 5)**								
		Month 4	5.0 (4.0; 5.0)	24	4.0 (3.0; 5.0)	25	5282.50	.03	0.58 (0.51-0.65)
		Month 9	4.0 (30; 5.0)	20	4.0 (3.0; 5.0)	15	1973.50	.15	0.57 (0.47-0.66)
	**Satisfaction with coaching (scale 1 to 5)**								
		Months 0-4	5.0 (4.0; 5.0)	24	4.0 (4.0; 5.0)	25	1923.50	<.001	0.66 (0.58-0.73)
		Months 4-9	4.50 (3.25; 5.0)	24	5.0 (4.0; 5.0)	25	938.0	.33	0.55 (0.45-0.67)
		Months 0-9	5.0 (4.0; 5.0)	24	4.0 (4.0; 5.0)	25	5729.50	.03	0.58 (0.51-0.65)

^a^Vargha-Delaney *A* measure of stochastic superiority for effect size estimation. Limits for interpretation: 0.56 (small), 0.64 (medium), 0.71 (large). Between-group differences are observed when the lower bound of the 95% CI is >0.5 [69].

^b^The scoring of the index is divided into 4 categories with the following interpretations: exhaustion (score −4 or less), high-risk (score −3.9 to −2.5), at risk (score −2.4 to −1.0), and good (score −0.9–0.0).

^c^N/A: not applicable.

^d^Range is not applicable (dropout attrition describes how many people dropped out of the study).

**Table 5:** Changes in well-being over time.

Outcome, group, and period				Change					N+ (Sign test)			*P* value (exact)			Â^a^
				Median (25th; 75th)		n									
**Mental well-being**															
	**Research**														
		Months 0-4	0.54 (0.12; 1.33)		16		13.0			.02			0.81		
		Months 0-9	0.98 (0.29; 2.33)		21		18.0			.001			0.86		
	**Control**														
		Months 0-4	2.11 (0.43; 4.60)		22		20.0			<.001			0.91		
		Months 0-9	2.41 (1.01; 4.56)		19		18.0			<.001			0.95		

^a^Vargha-Delaney *A* measure of stochastic superiority for effect size estimation. Limits for interpretation: 0.56 (small), 0.64 (medium), 0.71 (large) [69].

**Table 6:** Distribution of WorkOptimum value categories at different time points for both groups (n=49).

Time point		Research (n=24), n (%)	Control (n=25), n (%)
**Month 0**			
	Exhaustion	5 (21)	7 (28)
	High risk	6 (25)	4 (16)
	At risk	9 (38)	12 (48)
	Good	4 (17)	2 (8)
	Missing	0 (0)	0 (0)
**Month 4**			
	Exhaustion	2 (8)	3 (12)
	High risk	1 (4)	0 (0)
	At risk	5 (20)	9 (36)
	Good	8 (33)	10 (40)
	Missing	8 (33)	3 (12)
**Month 9**			
	Exhaustion	2 (8)	1 (4)
	High risk	0 (0)	2 (8)
	At risk	2 (8)	4 (16)
	Good	17 (79)	12 (48)
	Missing	3 (12.5)	6 (24)

### Time Use of Coaches

The total time use of coaches during the 9-month coaching period was not statistically significantly different between the 2 groups (366.0 vs 343.0 minutes, Â=0.60, 95% CI 0.33-0.85; *P*=.48; Table 4). However, the mean preparation time per coaching call was considerably higher in the research group (Â=0.90, 95% CI 0.75-1.0; *P*=.001). The mean duration per call did not differ between the groups (Â=0.59, 95% CI 0.37-0.80; *P*=.40). Regarding personal coaching messages, the coaches sent a total of 60 and 102 personal coaching messages to the research group participants during the intensive and maintenance phases, respectively. The median number of messages sent per participant was 2.0 (range 0-6) for the intensive and 4.0 (range 0-19) for the maintenance phase. Therefore, the estimated median time used for coaching messaging per participant was 10.0 minutes in the intensive phase and 40.0 minutes in the maintenance phase.

### Adherence

The dropout attrition rates differed between the study groups: 13% (3/24) in the research group and 24% (6/25) in the control group.

The use adherence differed somewhat between the study groups. Owing to the higher dropout attrition in the control group, the mean proportion of realized coaching calls was lower compared with the research group (86% vs 92%). There were no between-group statistical differences in task performance frequency for either phase (Â=0.50, 95% CI 0.42-0.57; *P*=.95, and Â=0.54, 95% CI 0.45-0.63; *P*=.37). The research group performed the tasks with slightly better diligence (Â=0.58, 95% CI 0.51-0.65; *P*=.03) during the intensive phase, but no differences were observed in the maintenance phase (Â=0.57, 95% CI 0.47-0.66; *P*=.15).

### Satisfaction With Coaching

During the intensive phase (months 0-4), the research group was moderately more satisfied with the coaching program than the control group (Â=0.66, 95% CI 0.58-0.73; *P*<.001). During the maintenance phase (months 4-9), satisfaction did not differ between the groups (Â=0.55, 95% CI 0.45-0.67; *P*=.33). In general, participant satisfaction was high throughout the coaching program in both groups (Table 4).

## Discussion

The aim of this study was to investigate whether technology-assisted telephone intervention is feasible for increasing well-being or decreasing the time use of coaches while maintaining participants’ adherence and satisfaction compared with traditional telephone intervention in an occupational health care setting.

### Principal Findings

The technology-assisted telephone intervention was similarly effective in increasing well-being as traditional telephone coaching while having better adherence in 2 of the 4 metrics (lower dropout rate and higher adherence to calls) and higher satisfaction during the intensive phase. However, technology-assisted telephone coaching was unable to demonstrate savings in the time use of coaches. Therefore, the intervention is feasible, but some modifications are needed before moving on to a large-scale RCT.

The similar well-being improvements in both groups might be due to the similarities in coaching content and having human contact in both groups. In addition, it might be due to the similar baseline well-being state being only at the “at risk” level for both groups. If a person is in a poorer condition, it might be more difficult to improve mental well-being with technology or less intensive coaching, but this remains to be studied among people with poorer well-being. It is noteworthy that for the technology-assisted group, the improvement in well-being holds, although the coaching calls were replaced with personal coaching messages during the maintenance phase. Therefore, it seems feasible to replace at least some of the coaching calls with messages.

Although the total time use of coaches was similar for the 2 groups, the coaches spent more time preparing for the coaching calls of the technology-assisted group participants. The potential reason for this could be that the used technology components generated additional information on the participant’s situation, which the coaches had to review before the coaching calls (Firstbeat well-being analysis, HRS’s behavior change needs analysis and coaching task recommendations, and participants’ progress in the Movendos Coaching Platform). However, the use of technology for providing a comprehensive picture of participants’ situations (needs, motivation, and progress) should be considered an advantage, as this supports coaches in making better decisions when identifying suitable behavior change goals and activities for the coaches; this type of information could also help coaches gain a better understanding of coaches’ motivation levels and the appropriate means to motivate them. Therefore, using analytical technological tools could lead to improved coaching quality and intervention effectiveness. This line of thinking is also supported by the observation that participants in the technology-assisted group reported higher satisfaction with coaching during the intensive phase than did the traditional intervention group. In addition, the used technology and technology-assisted coaching process were new to the coaches. Learning to use the new tools effectively as part of their coaching process must have taken extra time, while following the familiar telephone coaching process was obviously more efficient. There will be potential to enhance the use efficiency of new technology as they become familiar to end users.

The technology-assisted group was more persistent at staying in the intervention until the end and adhering to the coaching calls considering the dropout attrition rate was higher in the fully human intervention group. This might be due to the generally less effort needed from the technology-assisted group participants because there were no coaching calls in the maintenance phase. Perhaps it is easier to adhere to the lower number of calls. However, after the intensive phase, the technology-assisted group had a higher dropout rate than the traditional intervention group, but this difference was due to only 1 person. Moving to another town was the reason for 1 of 3 dropouts. The satisfaction for the 2 other dropouts was good and, therefore, does not explain the dropout. The task performance frequency was similar between the 2 groups. Better diligence in the technology-assisted group in the intensive phase could have been because of extensive analysis of behavior change needs at the beginning, which might have increased the personal understanding of why the tasks were important, thus leading to higher levels of diligence in performing them.

The higher levels of satisfaction for the technology-assisted group in the intensive phase might be explained by the technology providing the coaches with a deeper understanding of participants’ situations and needs, which in turn may have facilitated coaches to provide the right kind of support. From this perspective, technology was fulfilling one of its goals in creating a comprehensive picture of the participant and enhancing coaching quality. However, satisfaction did not differ between the 2 groups during the maintenance phase, which is a good result because the coaching took place via messaging instead of phone calls for the technology-assisted group during this phase.

It is also interesting that 86% (42/49) of participants chose a task related to physical activity as part of the coaching plan. There are several potential reasons for this finding. One reason could be that many of the participants were employed by the health care sector and, hence, had knowledge of the basic principles of a healthy lifestyle. Coaches noted that participants commonly felt that they did not have enough time for physical activity. In Finnish culture, it is typical to think that exercise is a medicine for almost anything. Furthermore, the Fitbit wrist device offered to the technology-assisted group might have encouraged the focus on physical activity, as it enabled easy and motivating self-monitoring of activity.

### Comparison With Previous Studies

Our findings strengthen the evidence for technology-assisted human interventions being equally effective as traditional human interventions. However, the majority of the studies compared interventions with a different level of human-technology involvement from ours, where human coaching was partly or entirely replaced by technology.

Only 1 study considered the effectiveness of an intervention similar to this study, but it was compared with a fully technological intervention [23]. In this study, the technology-assisted intervention supported the coaches by providing comprehensive knowledge regarding the participant’s situation and was used as communication media, but it did not provide coaching without the coach.

Our findings could not confirm earlier claims of saving time with technology use [20]. However, earlier approaches were also different from this study in the way that technology usually replaced or automated coaching tasks. This study, however, provides more information on the coaches’ time use for the preparation of coaching calls and the actual call durations, which helps target changes to the intervention so that it might save time.

Adherence in both groups in terms of dropout rate (13% and 24%) was good. The adherence to earlier well-being interventions varied significantly. In another technology-assisted human (physical activity) intervention study, adherence to the intervention was 28.4% (dropout rate was 71.6%) [23]. In human-assisted technological stress management interventions, adherence varied between 69.7% and 96% [31,71,72] and, in fully technological interventions, it varied between 25% and 90.6% [23,30,31]. Thus, our findings indicate a high level of adherence.

This study provides new knowledge on satisfaction with stress management technology-assisted human interventions, showing that interventions with less intensive human involvement can lead to similar or even better participant satisfaction than more intensive human involvement.

### Limitations

There were several limitations to this study. One limitation is the narrow approximation of costs based on the time use of coaches. For a comprehensive cost-effectiveness study, costs should be studied more widely from both the care provider and societal perspectives. In addition, the technology itself incurs costs that have to be considered.

Considering the study was not blinded to the participants and they knew the intervention of interest and the intervention they were participating in, it is possible that participants may have sought additional help, for example, from occupational health care to which all participants had access. Providing sufficient information is the result of balancing a valid research setting and ethics. Moreover, participants in the traditional telephone intervention group were more highly educated, which could have a positive impact on their motivation and ability to seek additional help. These issues may have affected our results, perhaps by improving the well-being of the control group.

As the used technology components and technology-assisted coaching process were new to the coaches, the study might not provide realistic results on the time use of coaches. Our study also showed that there can be many problems with human-reported metrics. There can be challenges in advising procedures, which in our case caused a misunderstanding between researchers and coaches regarding the proper procedures to record the time used for coaching. In addition, there is a risk of measurement errors when relying on self-reporting instead of objective measurements. In addition, the data collection metrics for adherence had to be improved during the pilot because the measurement scales for evaluating adherence during the intensive phase turned out to be ambiguous. The recording of time used was also stated by the coaches to be too laborious. Future studies should ensure that time logging is easy, preferably even automatic, and that it is performed consistently throughout the study.

The sample size was small, which makes the results only preliminary and must be confirmed in a larger RCT. With a small number of participants, unexpected events during the study and participant-specific variations in the selected intervention areas may have influenced the study results. The sample was also quite biased, containing mostly middle-aged, highly educated women because of the recruitment methodology. The participants were recruited from the municipal sector (employees of the City of Oulu, Finland), where especially in education, health care and social sectors, highly educated women are in majority, so it is obvious that these women are also well represented in this study. In addition, women are generally more interested in their health and more eager to participate in studies [73]. For better generalizability of the results, it would be essential to recruit participants from a group that has more variety in terms of age, gender, and education level. This could be achieved using several occupational care providers that have customers from different industries (eg, also including the manufacturing industry, where most of the employees are men).

The results were obtained in the coaching environment in which phone calls were the primary means of coaching and may not be generalized to other forms of coaching. In addition, coaching can be implemented in various ways, which makes proper comparison of different interventions or studies difficult. Individual situations and health statuses vary considerably between people, and here the participants had moderate baseline well-being. Therefore, it is difficult to generalize any results from this study, and the results only hold for this particular sample, set of tools, and processes. In addition, in the absence of a no-treatment group, the role of other factors, such as the research setting itself, cannot be quantified.

### Implications

The feasibility of the technology-assisted intervention was compromised because of the inability to show a decrease in the time use of coaches. This may be due to an inefficient coaching process or unfamiliarity with the used technology, subjective (error-prone) data collection methods, and the small sample size.

It is expected that the process can save coaches’ time once the technology and its optimal use practices have been honed and become more of a routine. Therefore, coaching technology should be used for some time before starting a larger RCT. Studies should also explore which coaching activities could be further automated to maintain the effects but decrease the time use of coaches. During action planning, it is important to provide concise reports that are easy and quick for coaches to read and understand. In addition, using templates for messaging can be helpful in decreasing the time use of coaches.

There is a need for reliable objective data collection methods for the time use of coaches. It would be ideal if the data could be collected automatically, and with digital interventions, this becomes possible. Technology can support research data collection by prompting the coach after each coaching event to note how much time was used for preparation and by automatically recording the coaching call durations and writing messages, for example.

The effect size and adherence rates allowed for an estimation of the number of participants to be enrolled in the fully powered RCT. A small effect size (Â=0.56) was obtained for the difference in well-being between the groups in the end. Thus, it would be required to enroll 125 (58+67) participants (considering 13%-24% dropouts; power=0.80; significance level, α=.05) in a fully powered 2-arm RCT to study the difference in effects on well-being [74].

### Conclusions

The studied technology-assisted telephone intervention is feasible with some modifications by showing similar preliminary effectiveness as the traditional telephone intervention, better satisfaction, and better adherence in 2 out of 4 adherence indicators. However, because it did not reduce the time use of coaches, it requires modifications before conducting a large-scale RCT. On the one hand, these adjustments should include adding features to the technology components to support further the work of coaches, for instance, by providing the available data regarding participants’ situation in a more digested format that is fast to comprehend and providing templates for personal coaching messages. On the other hand, the protocol should be improved by recruiting more participants, using objective and automatic time-tracking methods and starting the study once the coaches have established a routine of using the technology components as part of the coaching process. Technology seems promising in terms of facilitating less resource-intensive personal coaching by replacing some coaching calls with coaching messages, but further studies are required to confirm this.

## Appendix

Multimedia Appendix 1 Mental well-being—WorkOptimum.

Multimedia Appendix 2 Use adherence—frequency of and diligence in performing the tasks.

Multimedia Appendix 3 CONSORT-eHEALTH checklist (V 1.6.1).

## Abbreviations

HRS: health recommender system
HRV: heart rate variability
RCT: randomized controlled trial
