# Supplementary material for: A Technology-Assisted Telephone Intervention for Work-Related Stress Management: Pilot Randomized Controlled Trial
Source: J Med Internet Res. 2022 Jul 13;24(7):e26569. doi: 10.2196/26569 (PMC9330204; doi:10.2196/26569)
Supplement: Multimedia Appendix 1 [file jmir_v24i7e26569_app1.docx]

# APPENDIX A: Mental Well-Being - WorkOptimum ®

WorkOptimum consists of 17 items. There are three sub-indexes (recovery, resource balance, and workload) that are used for calculating the fourth, the WorkOptimum index (Ahveninen et al. 2014). The indexes in the three sub-categories can have any values in the infinity and they have the following interpretations: “Very poor/negative” (score less than -4), “Somewhat poor/negative” (score -3 to -1), “Sufficient/mediocre” (score 0 to 2), “Good” (score 3 to 5), and “Excellent” (score over 5). The WorkOptimum index itself receives values from the negative infinity to zero, and the decline in cognitive ability and decrease in mental well-being are categorized by the index as “exhaustion” (score -4 or less), “high-risk” (score -3.9 to -2.5), “at risk” (score -2.4 to -1), and “good” (score -0.9 to 0).

WorkOptimum items have been constructed from various other scales and questionnaires, e.g., Effort-Reward Imbalance (ERI), 13-item Sense of Coherence (SOC-13), 16-item Maslach Burnout Inventory (MBI-16), 15-item Bergen Burnout Indicator (BBI-15), Työkykyindeksi (English: workability index)(TKI) questionnaire, Need for Recovery (NFR), Beck Depression Inventory (BDI), 90-item Symptom Check List (SCL-90), and Voimavarat Työssä (English: resources at work) (VVT) questionnaire. There is an English translation of WorkOptimum in use in Finland in Terveystalo (private healthcare provider). The translations below follow the English version in use. However, some reply options vary in order or in terms of the scale division in the questions indicated below.

Table 1. WorkOptimum® questionnaire

| **#** | **Item** | **Scale** |
| --- | --- | --- |
| 1 | Length of your work week during the last two months, including work done at home | Less than 40 h  40­–44 hours per week  45–50 hours per week  More than 55 hours per week |
| 2 | Time pressure caused by your workload is | Minor  Occasional workload peaks  Almost continuous, does not burden  Continuous and somewhat burdensome  Continuous and very burdensome |
| 3 | Things that bring you satisfaction in your work are present despite possible distractions *^1^* | Very little  Sort of  Sufficiently  Very well |
| 4 | A certain lack of pleasure (joy of working) has been a distinct feature of my work for at least one to two months ^2^ | Completely disagree  Disagree  Agree  Completely agree |
| 5 | I have symptoms, pains or illnesses that are either caused by work or get worse because of work | No  Yes, minor  Yes, major |
| 5b) | Which of the following symptoms do you have? | None  Sleeping problems  Fatigue  Pains  *Back pains ^2^*  *Neck pains ^2^*  *Upper limb problems ^2^*  *Headaches ^2^*  *Lower limb problems ^2^*  Memory problems  Concentration difficulties  *Low mood ^2^*  Other_____________________ |
| 6 | I can easily get started even with demanding tasks *^1^* | Completely disagree  Disagree  Agree  Completely agree |
| 7 | How fresh do you feel after a workday? | 10-point scale, 1 “Very tired” and 10 “Very fresh” |
| 8 | How long into the future will your current resources last with your current way of working? *^3^* | Less than 2 months  6 months  1­–2 years  At least 5 years  Throughout my career / until retirement |
| 9 | Have you had trouble with falling asleep, waking up during the night without being able to fall asleep again or experienced shortened sleep? | Once or less a week  Twice a week  3–5 times a week  Daily |
| 10 | When was the last time I have had a sustained feeling of being energetic? *^4^* | Yesterday  One of these days  1-2 weeks ago  About one month ago  2-4 months ago  6 months ago  More than 6 months ago |
| 11 | For a person of my age, my physical health is | Tolerable  Below Average  Average  Good  Excellent |
| 12 | My mental resources are | Completely exhausted  Slightly overused  Ordinary  Good  Excellent |
| 13 | I manage not to think about work-related problems in my spare time. | Completely Disagree  Disagree  Agree  Completely Agree |
| 14 | I get irritated by other people’s behavior more easily than before. | Completely Disagree  Disagree  Agree  Completely Agree |
| 15 | To counterbalance my work, I do relaxing things or stick to my hobbies. | Completely Disagree  Disagree  Agree  Completely Agree |
| 16 | My interest in sex has decreased significantly: “I want to, but I’m too tired.” | Completely Disagree  Disagree  Agree  Completely Agree |
| 17 | I drink alcohol more frequently or consume it more than usually. | Completely Disagree  Disagree  Agree  Completely Agree |

[1] Answers in reverse order in the official English version

[2] Answer option only in the official English version

[3] Answer options in the official English version: Less than 2 months, 3–6 months, 7–11 months, 1–2 years, 3–5 years, More than 5 years, Throughout my career/until retirement (choose this if you have more than 10 years to retirement)

[4] Answer options in the official English version: Yesterday – 2–3weeks ago, About 1 month ago, More than 2 months ago, 3–6 months ago, More than half a year ago

**Reference**

Ahveninen, H. Rintala, J., Ollikainen, J., Suohonen, J., and Arola, H. Työoptimi-kysely auttaa tunnistamaan työssäkäyvän kognitiivisia ja muistioireita [*WorkOptimum questionnaire helps to detect memory and cognitive symptoms of the working people*]. Suomen Lääkärilehti, 4, 2014.
