# Supplementary material for: A Technology-Assisted Telephone Intervention for Work-Related Stress Management: Pilot Randomized Controlled Trial
Source: J Med Internet Res. 2022 Jul 13;24(7):e26569. doi: 10.2196/26569 (PMC9330204; doi:10.2196/26569)
Supplement: Multimedia Appendix 2 [file jmir_v24i7e26569_app2.docx]

# APPENDIX B: Usage Adherence – Frequency of and Diligence in Performing the Task

## Data Collection Form for Coaches (Intensive Phase)

This questionnaire was created by the researchers for tracking participant adherence to the coaching. Coaches filled it in after each coaching call based on the interview during the intensive phase.

Table 1. Adherence to coaching (free translations by a researcher).

| **Questions** | 1. How actively did the customer perform the coaching task? | | | 2. Customer performed the coaching task carefully | 3. If the customer performed the task less/frequently or incompletely/partly (answer to question 1 or 2 is 3 or less) |
| --- | --- | --- | --- | --- | --- |
|  | 1a) Customer performed the task less than agreed | 1b) Customer performed the task more frequently than agreed | |  |  |
| **The name of the task** | *Scoring:*   \| *Strongly disagree* \|  \| *Neither disagree nor agree* \|  \| *Strongly agree* \| *Undecided* \| \| --- \| --- \| --- \| --- \| --- \| --- \| \| *1* \| *2* \| *3* \| *4* \| *5* \| *0* \| | | | | *Answer options 1 – 15 (see below)* |
|  | 1 2 3 4 5 0 | | 1 2 3 4 5 0 | 1 2 3 4 5 0 |  |
|  | 1 2 3 4 5 0 | | 1 2 3 4 5 0 | 1 2 3 4 5 0 |  |
|  | 1 2 3 4 5 0 | | 1 2 3 4 5 0 | 1 2 3 4 5 0 |  |
|  | 1 2 3 4 5 0 | | 1 2 3 4 5 0 | 1 2 3 4 5 0 |  |
|  | 1 2 3 4 5 0 | | 1 2 3 4 5 0 | 1 2 3 4 5 0 |  |

**Answer options for the follow-up questions**

Reasons for low activity or carelessness in performing the tasks / Syitä vähäiselle aktiivisuudelle tai huolimattomuudelle tehtävien teossa:

1. The task was too difficult / Tehtävä oli liian vaikea
2. The task took too long or performing it was too cumbersome / Tehtävä vei liikaa aikaa tai sen tekeminen oli liian työlästä
3. It was difficult to fit the task in with everyday life / Tehtävää oli hankala sovittaa arkeen
4. The task felt pointless / Tehtävä tuntui hyödyttömältä
5. The task didn’t feel necessary anymore because the customer had achieved the goal that was in the background / Tehtävä ei tuntunut enää tarpeelliselta, sillä asiakas oli jo mielestään saavuttanut tehtävän taustalla olevan tavoitteensa
6. Performing the task was not nice or pleasant / Tehtävän tekeminen ei ollut mukavaa taikka miellyttävää
7. Fatigue or lack of energy / Väsymys tai energian puute
8. Other reason, what? / Muu syy, mikä? *(kirjoita taulukkoon)*
9. Undecided/ En osaa sanoa

Reasons for performing the task more actively / Syitä sovittua aktiivisemmalle tehtävien teolle:

1. The task felt easy / Tehtävä tuntui helpolta
2. The task was pleasant / Tehtävä oli mieluisa
3. The task was useful / Tehtävästä oli hyötyä
4. The task felt inefficient / Tehtävä tuntui tehottomalta
5. Other reason, what? / Muu syy, mikä? *(kirjoita taulukkoon)*
6. Undecided */* En osaa sanoa

## Questionnaire for the Participants (Maintenance Phase)

This electronic questionnaire was used in the maintenance phase to collect data on the adherence to coaching during the maintenance phase.

Table 2. Adherence to coaching questionnaire (maintenance phase, free translations by a researcher).

| **Item** | **Scale** |
| --- | --- |
| 1. Name of the coaching task or free description |  |
| 2. How actively did you perform the coaching task? | 1. Much less than agreed  2. Slightly less than agreed  3. As agreed  4. Slightly more frequently than agreed  5. Much more frequently than agreed  EOS. Undecided |
| 2b. Reason for performing the task less | 1. The task was too difficult  2. The task took too much time or performing it was too cumbersome  3. It was difficult to fit the task in with everyday life  4. The task felt pointless  5. The task didn’t feel necessary anymore because the customer had achieved the goal that was in the background  6. Performing the task was not nice or pleasant  7. Fatigue or lack of energy  8. Other reason, what?______________________  EOS. Undecided |
| 2c. Reasons for performing the task more frequently | 1. The task felt easy  2. The task was pleasant  3. The task was useful  4. The task felt inefficient  5. Other reason, what? ______________________  EOS. Undecided |
| 3. I performed the coaching task carefully | 1. Strongly disagree  2. Slightly disagree  3. Nor disagree nor agree  4. Slightly agree  5. Strongly agree  EOS. Undecided |
| 3b. Reason for performing the task incompletely/partly | 1. The task was too difficult  2. The task took too much time or performing it was too cumbersome  3. It was difficult to fit the task in with everyday life  4. The task felt pointless  5. The task didn’t feel necessary anymore because the customer had achieved the goal that was in the background  6. Performing the task was not nice or pleasant  7. Fatigue or lack of energy  8. Other reason, what? ______________________  EOS. Undecided |
