# Supplementary material for: A Technology-Assisted Telephone Intervention for Work-Related Stress Management: Pilot Randomized Controlled Trial
Source: J Med Internet Res. 2022 Jul 13;24(7):e26569. doi: 10.2196/26569 (PMC9330204; doi:10.2196/26569)
Supplement: Multimedia Appendix 3 [file jmir_v24i7e26569_app3.pdf]

CONSORT-EHEALTH (V 1.6.1) - Submission/Publication Form
The CONSORT-EHEALTH checklist is intended for authors of randomized trials evaluating web-based and Internet-based applications/interventions, including mobile interventions, electronic games (incl multiplayer games), social media, certain telehealth applications, and other interactive and/or networked electronic applications. Some of the items (e.g. all subitems under item 5 - description of the intervention) may also be applicable for other study designs.

The goal of the CONSORT EHEALTH checklist and guideline is to be
a) a guide for reporting for authors of RCTs,
b) to form a basis for appraisal of an ehealth trial (in terms of validity)

CONSORT-EHEALTH items/subitems are MANDATORY reporting items for studies published in the Journal of Medical Internet Research and other journals / scientific societies endorsing the checklist.

Items numbered 1., 2., 3., 4a., 4b etc are original CONSORT or CONSORT-NPT (non-pharmacologic treatment) items.
Items with Roman numerals (i., ii, iii, iv etc.) are CONSORT-EHEALTH extensions/clarifications.

As the CONSORT-EHEALTH checklist is still considered in a formative stage, we would ask that you also RATE ON A SCALE OF 1-5 how important/useful you feel each item is FOR THE PURPOSE OF THE CHECKLIST and reporting guideline (optional).

Mandatory reporting items are marked with a red *.
In the textboxes, either copy & paste the relevant sections from your manuscript into this form - please include any quotes from your manuscript in QUOTATION MARKS,
or answer directly by providing additional information not in the manuscript, or elaborating on why the item was not relevant for this study.

YOUR ANSWERS WILL BE PUBLISHED AS A SUPPLEMENTARY FILE TO YOUR PUBLICATION IN JMIR AND ARE CONSIDERED PART OF YOUR PUBLICATION (IF ACCEPTED).
Please fill in these questions diligently. Information will not be copyedited, so please use proper spelling and grammar, use correct capitalization, and avoid abbreviations.

DO NOT FORGET TO SAVE AS PDF _AND_ CLICK THE SUBMIT BUTTON SO YOUR ANSWERS ARE IN OUR DATABASE !!!

Citation Suggestion (if you append the pdf as Appendix we suggest to cite this paper in the caption):
Eysenbach G, CONSORT-EHEALTH Group
CONSORT-EHEALTH: Improving and Standardizing Evaluation Reports of Web-based and Mobile Health Interventions
J Med Internet Res 2011;13(4):e126
URL: http://www.jmir.org/2011/4/e126/
doi: 10.2196/jmir.1923
PMID: 22209829
*Pakollinen

Your name *
Salla Muuraiskangas

Primary Affiliation (short), City, Country *
VTT Technical Research Centre of Finland Ltd., Oulu, Finland

Your e-mail address *
salla.muuraiskangas@vtt.fi

Title of your manuscript *
Technology-Assisted Telephone Intervention for Work-Related Stress Management: Pilot Randomized Controlled Trial

Name of your App/Software/Intervention *
Movendos

Evaluated Version (if any)
e.g. "V1", "Release 2017-03-01", "Version 2.0.27913"
v1.27

Language(s) *
Finnish

URL of your Intervention Website or App
e.g. a direct link to the mobile app on app in appstore (itunes, Google Play), or URL of the website. If the intervention is a DVD or hardware, you can also link to an Amazon page.

URL of an image/screenshot (optional)

Accessibility *
Can an enduser access the intervention presently?

- access is free and open
- access only for special usergroups, not open
- access is open to everyone, but requires payment/subscription/in-app purchases
- **app/intervention no longer accessible**
- Muu:

Primary Medical Indication/Disease/Condition *
Mental well-being

Primary Outcomes measured in trial *
Mental well-being, time use of the coaches

Secondary/other outcomes
Are there any other outcomes the intervention is expected to affect?

Adherence, satisfaction with coaching

Recommended "Dose" *
What do the instructions for users say on how often the app should be used?
- Approximately Daily
- Approximately Weekly
- **Approximately Monthly**
- Approximately Yearly
- "as needed"
- Muu:

Approx. Percentage of Users (starters) still using the app as recommended after 3 months *
- **unknown / not evaluated**
- 0-10%
- 11-20%
- 21-30%
- 31-40%
- 41-50%
- 51-60%
- 61-70%
- 71%-80%
- 81-90%
- 91-100%
- Muu:

Overall, was the app/intervention effective? *
- yes: all primary outcomes were significantly better in intervention group vs control
- partly: SOME primary outcomes were significantly better in intervention group vs control
- **no statistically significant difference between control and intervention**
- potentially harmful: control was significantly better than intervention in one or more outcomes
- inconclusive: more research is needed
- Muu:

Article Preparation Status/Stage *
At which stage in your article preparation are you currently (at the time you fill in this form)
- not submitted yet - in early draft status
- not submitted yet - in late draft status, just before submission
- submitted to a journal but not reviewed yet
- **submitted to a journal and after receiving initial reviewer comments**
- submitted to a journal and accepted, but not published yet
- published
- Muu:

Journal *
If you already know where you will submit this paper (or if it is already submitted), please provide the journal name (if it is not JMIR, provide the journal name under "other")
- not submitted yet / unclear where I will submit this

- **Journal of Medical Internet Research (JMIR)**
- JMIR mHealth and UHealth
- JMIR Serious Games
- JMIR Mental Health
- JMIR Public Health
- JMIR Formative Research
- Other JMIR sister journal
- Muu:

Is this a full powered effectiveness trial or a pilot/feasibility trial? *
- **Pilot/feasibility**
- Fully powered

Manuscript tracking number *
If this is a JMIR submission, please provide the manuscript tracking number under "other" (The ms tracking number can be found in the submission acknowledgement email, or when you login as author in JMIR. If the paper is already published in JMIR, then the ms tracking number is the four-digit number at the end of the DOI, to be found at the bottom of each published article in JMIR)
- no ms number (yet) / not (yet) submitted to / published in JMIR
- **Muu: #26569**

TITLE AND ABSTRACT

1a) TITLE: Identification as a randomized trial in the title

1a) Does your paper address CONSORT item 1a? *
I.e does the title contain the phrase "Randomized Controlled Trial"? (if not, explain the reason under "other")
- **yes**
- Muu:

1a-i) Identify the mode of delivery in the title
Identify the mode of delivery. Preferably use “web-based” and/or “mobile” and/or “electronic game” in the title. Avoid ambiguous terms like “online”, “virtual”, “interactive”. Use “Internet-based” only if Intervention includes non-web-based Internet components (e.g. email), use “computer-based” or “electronic” only if offline products are used. Use “virtual” only in the context of “virtual reality” (3-D worlds). Use “online” only in the context of “online support groups”. Complement or substitute product names with broader terms for the class of products (such as “mobile” or “smart phone” instead of “iphone”), especially if the application runs on different platforms.
subitem not at all important 1 2 3 4 5 essential

Does your paper address subitem 1a-i? *
Copy and paste relevant sections from manuscript title (include quotes in quotation marks "like this" to indicate direct quotes from your manuscript), or elaborate on this item by providing additional information not in the ms, or briefly explain why the item is not applicable/relevant for your study
Yes. "Technology-assisted telephone Intervention". Intervention main delivery was through telephone coaching, which was complemented e.g. by profiling to support the coach and messaging via web-based coaching platform.

1a-ii) Non-web-based components or important co-interventions in title
Mention non-web-based components or important co-interventions in title, if any (e.g., "with telephone support").
subitem not at all important 1 2 3 4 5 essential

Does your paper address subitem 1a-ii?
Yes. "Technology-assisted telephone Intervention"

1a-iii) Primary condition or target group in the title
Mention primary condition or target group in the title, if any (e.g., "for children with Type I Diabetes") Example: A Web-based and Mobile Intervention with Telephone Support for Children with Type I Diabetes: Randomized Controlled Trial
subitem not at all important 1 2 3 4 5 essential

Does your paper address subitem 1a-iii? *
Yes. " Work-Related Stress "

1b) ABSTRACT: Structured summary of trial design, methods, results, and conclusions

NPT extension: Description of experimental treatment, comparator, care providers, centers, and blinding status.
1b-i) Key features/functionalities/components of the intervention and comparator in the METHODS section of the ABSTRACT
Mention key features/functionalities/components of the intervention and comparator in the abstract. If possible, also mention theories and principles used for designing the site. Keep in mind the needs of systematic reviewers and indexers by including important synonyms. (Note: Only report in the abstract what the main paper is reporting. If this information is missing from the main body of text, consider adding it)
subitem not at all important 1 2 3 4 5 essential

Does your paper address subitem 1b-i? *
Yes. " The coaching methodology was based on habit formation, motivational interviewing and the transtheoretical model. For the research group, technology supported both the coaches and participants in identifying behaviour change targets, setting the initial coaching plan, progress monitoring, and communication."

1b-ii) Level of human involvement in the METHODS section of the ABSTRACT
Clarify the level of human involvement in the abstract, e.g., use phrases like "fully automated" vs. "therapist/nurse/care provider/physician-assisted" (mention number and expertise of providers involved, if any). (Note: Only report in the abstract what the main paper is reporting. If this information is missing from the main body of text, consider adding it)
subitem not at all important 1 2 3 4 5 essential

Does your paper address subitem 1b-ii?
Yes. "For the research group, technology supported both the coaches and participants in identifying behaviour change targets, setting the initial coaching plan, progress monitoring, and communication. "

1b-iii) Open vs. closed, web-based (self-assessment) vs. face-to-face assessments in the METHODS section of the ABSTRACT

Mention how participants were recruited (online vs. offline), e.g., from an open access website or from a clinic or a closed online user group (closed usergroup trial), and clarify if this was a purely web-based trial, or there were face-to-face components (as part of the intervention or for assessment). Clearly say if outcomes were self-assessed through questionnaires (as common in web-based trials). Note: In traditional offline trials, an open trial (open-label trial) is a type of clinical trial in which both the researchers and participants know which treatment is being administered. To avoid confusion, use "blinded" or "unblinded" to indicated the level of blinding instead of "open", as "open" in web-based trials usually refers to "open access" (i.e. participants can self-enrol). (Note: Only report in the abstract what the main paper is reporting. If this information is missing from the main body of text, consider adding it)
subitem not at all important 1 2 3 4 5 essential

Does your paper address subitem 1b-iii?
Yes. "Participants were recruited online through a regional occupational health care provider, and randomized equally to a research (technology-assisted telephone intervention) and a control (traditional telephone intervention) group. " ..." Pilot outcome was intervention feasibility measured primarily by the self-assessed mental well-being (WorkOptimum index) and the self-reported time use of coaches, and secondarily by participants' adherence and satisfaction"

1b-iv) RESULTS section in abstract must contain use data
Report number of participants enrolled/assessed in each group, the use/uptake of the intervention (e.g., attrition/adherence metrics, use over time, number of logins etc.), in addition to primary/secondary outcomes. (Note: Only report in the abstract what the main paper is reporting. If this information is missing from the main body of text, consider adding it)
subitem not at all important 1 2 3 4 5 essential

Does your paper address subitem 1b-iv?
Yes. "Regarding adherence, the dropout rate was 12.50% (3/24) and 24.00% (6/25), and the mean adherence rate to coaching calls was 91.67% and 85.50% for the research and control groups, respectively "

1b-v) CONCLUSIONS/DISCUSSION in abstract for negative trials
Conclusions/Discussions in abstract for negative trials: Discuss the primary outcome - if the trial is negative (primary outcome not changed), and the intervention was not used, discuss whether negative results are attributable to lack of uptake and discuss reasons. (Note: Only report in the abstract what the main paper is reporting. If this information is missing from the main body of text, consider adding it)
subitem not at all important 1 2 3 4 5 essential

Does your paper address subitem 1b-v?
Yes. " The technology-assisted telephone intervention is feasible with some modifications before moving to a larger scale, as it had similar preliminary effectiveness as the traditional telephone intervention, and the participants had better satisfaction with, and similar or better adherence to the intervention, but it did not reduce the time use of coaches. "

INTRODUCTION

2a) In INTRODUCTION: Scientific background and explanation of rationale

2a-i) Problem and the type of system/solution

Describe the **problem** and the type of system/**solution** that is object of the study: intended as stand-alone intervention vs. incorporated in broader health care program? Intended for a particular patient **population**? **Goals** of the intervention, e.g., being more cost-effective to other interventions, replace or complement other solutions? (Note: Details about the intervention are provided in "Methods" under 5)
subitem not at all important 1 2 3 4 5 essential

Does your paper address subitem 2a-i? *
Yes. "Work-related stress and its indirect consequences for physical, mental and social well-being are serious threats to public health."... "The previous research suggests that blended interventions have the potential to be effective, but cost-effectiveness studies are lacking for the stress management with technology-assisted human interventions (C2). Furthermore, adherence and satisfaction are important for evaluating the feasibility of interventions in more detail, and helping to refine their implementation for future large-scale RCT."..."The primary objective of this study was to investigate whether a technology-assisted telephone intervention (C2) for stress management is feasible for increasing participants' well-being or decreasing the time use of coaches, while maintaining participants' adherence and satisfaction compared to a traditional telephone intervention (C1) in an occupational health care setting"

2a-ii) Scientific background, rationale: What is known about the (type of) system
Scientific background, **rationale**: **What is known** about the (type of) system that is the object of the study (be sure to discuss the use of similar systems for other conditions/diagnoses, if appropiate), motivation for the study, i.e. what are the reasons for and what is the context for this specific study, from which stakeholder viewpoint is the study performed, potential impact of findings [2]. Briefly justify the choice of the comparator.
subitem not at all important 1 2 3 4 5 essential

Does your paper address subitem 2a-ii? *
Yes. " interventions are often supported with technology or they can be even fully digital. The use of technology can decrease human involvement and thus costs, which enables scaling up the intervention for a larger population and, therefore, holds a promise of a healthier population."…"This suggests that the optimal solution could be a blended intervention (C2-C4),maximizing the scalability and effectiveness."... "The previous research suggests that blended interventions have the potential to be effective, but cost-effectiveness studies are lacking for the stress management with technology-assisted human interventions (C2)."

2b) In INTRODUCTION: Specific objectives or hypotheses

Does your paper address CONSORT subitem 2b? *
Yes. "The primary objective of this study was to investigate whether a technology-assisted telephone intervention (C2) for stress management is feasible for increasing participants' well-being or decreasing te time use of coaches, while maintaining participants' adherence and satisfaction compared to a traditional telephone intervention (C1) in an occupational health care setting."

METHODS

3a) Description of trial design (such as parallel, factorial) including allocation ratio

Does your paper address CONSORT subitem 3a? *
Yes. "A non-blinded, parallel-group, two-arm pilot RCT of nine months was conducted in Oulu, Finland, to explore whether a technology-assisted telephone intervention for stress management is feasible for increasing mental well-being or decreasing time use of coaches, while maintaining

adherence and satisfaction." ..."The eligible participants were randomly allocated either to a research (technology-assisted telephone intervention) or control (traditional telephone intervention) group in a 1:1 ratio, using stratified block randomization."

3b) Important changes to methods after trial commencement (such as eligibility criteria), with reasons

Does your paper address CONSORT subitem 3b? *
No. There was no important changes to methods.

3b-i) Bug fixes, Downtimes, Content Changes
Bug fixes, Downtimes, Content Changes: ehealth systems are often dynamic systems. A description of changes to methods therefore also includes important changes made on the intervention or comparator during the trial (e.g., major bug fixes or changes in the functionality or content) (5-iii) and other “unexpected events” that may have influenced study design such as staff changes, system failures/downtimes, etc. [2].
subitem not at all important 1 2 3 4 5 essential

Does your paper address subitem 3b-i?
No. In our study there was no remarkable bug fixes, down times or content changes during the study.

4a) Eligibility criteria for participants

Does your paper address CONSORT subitem 4a? *
Yes. “. The eligibility criteria are presented in Table 1.”

4a-i) Computer / Internet literacy
Computer / Internet literacy is often an implicit “de facto” eligibility criterion - this should be explicitly clarified.
subitem not at all important 1 2 3 4 5 essential

Does your paper address subitem 4a-i?
No. Computer literacy was not inquired from the participants.

4a-ii) Open vs. closed, web-based vs. face-to-face assessments:
Open vs. closed, web-based vs. face-to-face **assessments**: Mention how participants were **recruited** (online vs. offline), e.g., from an open access website or from a clinic, and clarify if this was a purely **web-based trial, or there were face-to-face components** (as part of the intervention or for assessment), i.e., to what degree got the study team to know the participant. **In online-only trials**, clarify if participants were quasi-anonymous and whether having multiple identities was possible or whether technical or logistical measures (e.g., cookies, email confirmation, phone calls) were used to detect/prevent these.
subitem not at all important 1 2 3 4 5 essential

Does your paper address subitem 4a-ii? *
Yes. "The recruitment announcement was published on the intranet…, and in the magazines… occupational healthcare provider also recruited participants personally and via email. The registration to the study was conducted online via a link ... The registered employees received informed consent by regular mail... The signed consents were collected by a research partner, who provided the coaching service for the intervention. An electronic eligibility survey was sent to the

employees who returned the signed consents, and they were informed of the inclusion or exclusion via email."… "There were two interventions: technology assisted telephone intervention and traditional telephone intervention."

4a-iii) Information giving during recruitment
Information given during recruitment. Specify how participants were briefed for recruitment and in the informed consent procedures (e.g., publish the informed consent documentation as appendix, see also item X26), as this information may have an effect on user self-selection, user expectation and may also bias results.
subitem not at all important 1 2 3 4 5 essential

Does your paper address subitem 4a-iii?
Yes. "The registered employees received informed consent by regular mail, where information was provided regarding the two study groups, intervention, data collection, data processing, data privacy, research partners and contact details."

4b) Settings and locations where the data were collected

Does your paper address CONSORT subitem 4b? *
Yes. "employees of the City of Oulu"

4b-i) Report if outcomes were (self-)assessed through online questionnaires
Clearly report if outcomes were (self-)assessed through online questionnaires (as common in web-based trials) or otherwise.
subitem not at all important 1 2 3 4 5 essential

Does your paper address subitem 4b-i? *
Yes. "The pilot outcome was intervention feasibility measured primarily by the participants' self-assessed mental well-being and the total time use of the coaches for the complete coaching period and secondarily by participants' adherence to and satisfaction with coaching.
"…" For each coaching call, the coaches were asked to manually record the duration of the call and the time spent preparing for them (in minutes)."… "During the intensive phase, the coaches evaluated the task performance adherence (frequency and diligence)"…" During the maintenance phase, the participants self-assessed their task performance adherence for each coaching task via three electronic questionnaires administered at months 5, 7, and 9 (after calls #6–8 for the control group) (APPENDIX B)."… "Participants' satisfaction with coaching was assessed with one question at different phases of the trial. For the research group, during the intensive phase the statement was: "I was satisfied with the coaching call" and during the maintenance phase "I was satisfied with the coaching received via Movendos messages." For the control group the statement remained the same through the intervention, "I was satisfied with the coaching call."

4b-ii) Report how institutional affiliations are displayed
Report how institutional affiliations are displayed to potential participants [on ehealth media], as affiliations with prestigious hospitals or universities may affect volunteer rates, use, and reactions with regards to an intervention.(Not a required item – describe only if this may bias results)
subitem not at all important 1 2 3 4 5 essential

Does your paper address subitem 4b-ii?

5) The interventions for each group with sufficient details to allow replication, including how and when they were actually administered

5-i) Mention names, credential, affiliations of the developers, sponsors, and owners
Mention names, credential, affiliations of the developers, sponsors, and owners [6] (if authors/evaluators are owners or developer of the software, this needs to be declared in a “Conflict of interest” section or mentioned elsewhere in the manuscript).
subitem not at all important 1 2 3 4 5 essential

Does your paper address subitem 5-i?
Yes. "At the beginning of the intensive phase, the research group received Firstbeat heart rate variability (HRV) sensors and wore them for three days (Firstbeat Technologies Ltd., see more details below)"..."a Fitbit activity bracelet (Fitbit Inc.) was provided."... "Oiva stress management web service based on acceptance and commitment therapy (Oiva) was offered. "..."During the project, a web-tool, HRS, was developed to analyze participants’ behavior change need areas and to provide a personalized recommendation of suitable behaviour change actions, i.e., coaching tasks, based on the identified needs. "... "The research group used the Movendos coaching web service (v1.27) (Movendos Ltd.)" … “Ulla-Maija Junno works at Luona Hoiva Ltd. (previously Mawell Care Ltd.), which provided the coaching for the intervention, and Hannu Nieminen works at Movendos Ltd., which provided the web coaching platform for the intervention.”

5-ii) Describe the history/development process

Describe the history/development process of the application and previous formative evaluations (e.g., focus groups, usability testing), as these will have an impact on adoption/use rates and help with interpreting results.
subitem not at all important 1 2 3 4 5 essential

Does your paper address subitem 5-ii?

5-iii) Revisions and updating

Revisions and updating. Clearly mention the date and/or version number of the application/intervention (and comparator, if applicable) evaluated, or describe whether the intervention underwent major changes during the evaluation process, or whether the development and/or content was “frozen” during the trial. Describe dynamic components such as news feeds or changing content which may have an impact on the replicability of the intervention (for unexpected events see item 3b).
subitem not at all important 1 2 3 4 5 essential

Does your paper address subitem 5-iii?
Yes. "Movendos coaching web service (v1.27) (Movendos Ltd., www.movendos.com)"

5-iv) Quality assurance methods

Provide information on quality assurance methods to ensure accuracy and quality of information provided [1], if applicable.
subitem not at all important 1 2 3 4 5 essential

Does your paper address subitem 5-iv?
No. Experienced health coaches were responsible for delivering the intervention in practice. Apart from the Profiler, all the utilized technology components were in commercial use, and thus of high maturity level. Unit tests were used to verify that the Profiler worked as specified.

5-v) Ensure replicability by publishing the source code, and/or providing screenshots/screen-capture video, and/or providing flowcharts of the algorithms used

Ensure replicability by publishing the source code, and/or providing screenshots/screen-capture video, and/or providing flowcharts of the algorithms used. Replicability (i.e., other researchers should in principle be able to replicate the study) is a hallmark of scientific reporting.
subitem not at all important 1 2 3 4 5 essential

Does your paper address subitem 5-v?
No.

5-vi) Digital preservation

Digital preservation: Provide the URL of the application, but as the intervention is likely to change or disappear over the course of the years; also make sure the intervention is archived (Internet Archive, webcitation.org, and/or publishing the source code or screenshots/videos alongside the article). As pages behind login screens cannot be archived, consider creating demo pages which are accessible without login.
subitem not at all important 1 2 3 4 5 essential

Does your paper address subitem 5-vi?
No. The webcitation tool was not in use anymore.

5-vii) Access

Access: Describe how participants accessed the application, in what setting/context, if they had to pay (or were paid) or not, whether they had to be a member of specific group. If known, describe how participants obtained "access to the platform and Internet" [1]. To ensure access for editors/reviewers/readers, consider to provide a "backdoor" login account or demo mode for reviewers/readers to explore the application (also important for archiving purposes, see vi).
subitem not at all important 1 2 3 4 5 essential

Does your paper address subitem 5-vii? *
Yes. "The used technology was free for the participants and they used them in their everyday lives." Movendos and Oiva were used via internet and coaching via telephone. Fitbit required mobile app.

5-viii) Mode of delivery, features/functionalities/components of the intervention and comparator, and the theoretical framework

Describe **mode of delivery, features/functionalities/components of the intervention and comparator, and the theoretical framework** [6] used to design them (instructional strategy [1], behaviour change techniques, persuasive features, etc., see e.g., [7, 8] for terminology). This includes an in-depth **description of the content** (including where it is coming from and **who developed it**) [1]," whether [and how] it is **tailored** to individual circumstances and allows users to track their **progress** and **receive feedback**" [6]. This also includes a description of **communication delivery channels** and – if computer-mediated communication is a component – whether communication was synchronous or asynchronous [6]. It also includes information on presentation strategies [1], including page design principles, average amount of text on pages, presence of hyperlinks to other resources, etc. [1].
subitem not at all important 1 2 3 4 5 essential

Does your paper address subitem 5-viii? *

Yes. "The coaching was based on the **habit formation theory**"... "The tasks were related to different topics, namely sleep, physical activity, eating, alcohol consumption, smoking, recovery from stress, anxiety, personal values, workload management, quality of relationship, self-esteem, and weight management."… "The behavioral strategies were based on motivational interviewing (MI) and the transtheoretical model (TTM) of Prochaska [66,67]"" The main difference between the interventions was the number of telephone calls and the use of technology in coaching. The research group had **five coaching calls** during the intensive phase and **one** at the end."..." Web tools and wearables were used to support the identification of the participants' behaviour change targets, the creation of the initial intervention plan, progress monitoring, and communication."..."During the maintenance phase, the research group received **coaching only via the Movendos messages**."... "control group received **eight coaching calls**"

5-ix) Describe use parameters

Describe use parameters (e.g., intended "doses" and optimal timing for use). Clarify what instructions or recommendations were given to the user, e.g., regarding timing, frequency, heaviness of use, if any, or was the intervention used ad libitum.
subitem not at all important 1 2 3 4 5 essential

Does your paper address subitem 5-ix?
Yes. "The research group had five coaching calls during the intensive phase and one at the end. Control group had five coaching calls in the intensive phase and three in the maintenance phase." "The coaches were expected to send group messages to the research group once a month and personal coaching messages every two weeks, in addition to replying to any messages from the participants on a weekly basis. " "The control group received eight coaching calls in total: five in the intensive phase and three in the maintenance phase." The use of Movendos from participant side was when needed.

5-x) Clarify the level of human involvement

Clarify the level of human involvement (care providers or health professionals, also technical assistance) in the e-intervention or as co-intervention (**detail number and expertise of professionals involved**, if any, as well as "**type of assistance offered**, the timing and frequency of the support, how it is initiated, and the medium by which the assistance is delivered". It may be necessary to distinguish between the level of human involvement required for the trial, and the level of human involvement required for a routine application outside of a RCT setting (discuss under item 21 – generalizability).
subitem not at all important 1 2 3 4 5 essential

Does your paper address subitem 5-x?
Yes. "Coaching was performed by three coaches of the research partner, Mawell Care Ltd., "..."During the maintenance phase, the research group received coaching only via the Movendos messages. The coaches were expected to send group messages to the research group once a month and personal coaching messages every two weeks, in addition to replying to any messages from the participants on a weekly basis. Before sending messages, the coaches checked the participants' progress on Movendos. The coaching messages then focused on motivating them on tasks that were not progressing." [589]

5-xi) Report any prompts/reminders used

Report any **prompts/reminders** used: Clarify if there were prompts (letters, emails, phone calls, SMS) to use the application, what triggered them, frequency etc. It may be necessary to distinguish

between the level of prompts/reminders required for the trial, and the level of prompts/reminders for a routine application outside of a RCT setting (discuss under item 21 – generalizability).
subitem not at all important 1 2 3 4 5 essential

Does your paper address subitem 5-xi? *
Yes. "The research group used the Movendos coaching service for 1) communicating with the coach via messages (e.g., feedback), 2) progress monitoring, and 3) receiving reminders from the coach and setting reminders themselves if they so wished."

5-xii) Describe any co-interventions (incl. training/support)

Describe any **co-interventions** (incl**. training/support**): Clearly state any interventions that are provided in addition to the targeted eHealth intervention, as ehealth intervention may not be designed as stand-alone intervention. This includes training sessions and support [1]. It may be necessary to distinguish between the level of training required for the trial, and the level of training for a routine application outside of a RCT setting (discuss under item 21 – generalizability.
subitem not at all important 1 2 3 4 5 essential

Does your paper address subitem 5-xii? *
Yes. Movendos was the main supplementing intervention for the telephone coaching. Additionally: "Oiva stress management web service based on acceptance and commitment therapy"..."Oiva contains short few-minute exercises" " Firstbeat well-being analysis (Firstbeat Technologies Ltd.)[69] provided an analysis of the balance between activity and rest based on heartrate variability (HRV)"…"a Fitbit activity bracelet (Fitbit Inc.) was provided."

6a) Completely defined pre-specified primary and secondary outcome measures, including how and when they were assessed

Does your paper address CONSORT subitem 6a? *
Yes. " Mental well-being was assessed with the WorkOptimum® index, which is a measure of occupational health, aiming at detecting work-related cognitive decline and decrease in mental well-being before developing mental health problems (APPENDIX A) [56,57]"…" . The electronic questionnaire was administered to the participants at the baseline (month 0), and at the end of the intensive (month 4) and maintenance (month 9) phases." …" The total time use of coaches was tracked during the entire intervention regarding 1) *preparation time* for the coaching calls, 2) duration of the *coaching calls* (i.e., six calls for the research group and eight calls for the control group), and 3) the time spent on *writing personal coaching messages* to the research group. For each coaching call, the coaches were asked to manually record the duration of the call and the time spent preparing for them (in minutes). "… "Adherence was assessed by the dropout attrition, describing how many quit the intervention, and by the usage adherence (inversely non-usage attrition). The usage adherence comprised 1) the proportion of the realized coaching calls, 2) the frequency of performing the selected coaching tasks, and 3) diligence in performing the tasks. During the intensive phase, the coaches evaluated the task performance adherence (frequency and diligence) three times for the research group (during coaching calls #3–5) and four times for the control group (during calls #2–5) via a structured interview (APPENDIX B). For each coaching task, the following three items were assessed: "The client performed the task less frequently than agreed," "The client performed the task more frequently than agreed," and "The client performed the task with diligence," with a five-point Likert scale (1 = "Strongly disagree," 5 = "Strongly agree") also having the option "I don't know.""…" During the maintenance phase, the participants self-assessed their task performance adherence for each coaching task via three electronic questionnaires administered at months 5, 7, and 9 (after calls #6–8 for the control group)

(APPENDIX B).”…” Participants’ satisfaction with coaching was assessed with one question at different phases of the trial. For the research group, during the intensive phase the statement was: “I was satisfied with the coaching call” and during the maintenance phase “I was satisfied with the coaching received via Movendos messages.” For the control group the statement remained the same through the intervention, “I was satisfied with the coaching call.” “The satisfaction was asked at four timepoints (after calls #2–5 for the research group and after calls #3–5 for the control group) during the intensive phase and three times in the maintenance phase (after the calls #6–8 of the control group).”

6a-i) Online questionnaires: describe if they were validated for online use and apply CHERRIES items to describe how the questionnaires were designed/deployed
If outcomes were obtained through online questionnaires, describe if they were validated for online use and apply CHERRIES items to describe how the questionnaires were designed/deployed [9].
subitem not at all important 1 2 3 4 5 essential

Does your paper address subitem 6a-i?
No, online questionnaires were not validated with CHERRIES.

6a-ii) Describe whether and how “use” (including intensity of use/dosage) was defined/measured/monitored
Describe whether and how “use” (including intensity of use/dosage) was defined/measured/monitored (logins, logfile analysis, etc.). Use/adoption metrics are important process outcomes that should be reported in any ehealth trial.
subitem not at all important 1 2 3 4 5 essential

Does your paper address subitem 6a-ii?
Yes. "use" and adherence for following the overall intervention program. The Movendos use itself was not monitored. " The usage adherence comprised 1) the proportion of the realized coaching calls, 2) the frequency of performing the selected coaching tasks, and 3) diligence in performing the tasks "... " During the intensive phase, the coaches evaluated the task performance adherence (frequency and diligence) three times for the research group (during coaching calls #3–5) and four times for the control group (during calls #2–5) via a structured interview (APPENDIX B). "…” During the maintenance phase, the participants self-assessed their task performance adherence for each coaching task via three electronic questionnaires administered at months 5, 7, and 9 (after calls #6–8 for the control group) (APPENDIX B).”

6a-iii) Describe whether, how, and when qualitative feedback from participants was obtained
Describe whether, how, and when qualitative feedback from participants was obtained (e.g., through emails, feedback forms, interviews, focus groups).
subitem not at all important 1 2 3 4 5 essential

Does your paper address subitem 6a-iii?
No. Qualitative feedback was collected with electronic questionnaires and naturally the coaches heard something. The qualitative data is not included in the analyses.

6b) Any changes to trial outcomes after the trial commenced, with reasons

Does your paper address CONSORT subitem 6b? *
No. There were changes made to the outcomes after the trial commenced.

7a) How sample size was determined

NPT: When applicable, details of whether and how the clustering by care provides or centers was addressed

7a-i) Describe whether and how expected attrition was taken into account when calculating the sample size
Describe whether and how expected attrition was taken into account when calculating the sample size.
subitem not at all important 1 2 3 4 5 essential

Does your paper address subitem 7a-i?
Yes. "The available coaching resources defined how many participants could be enrolled to the study. At the time of the study, the participating coaching service provider employed three coaches, who could use on average 20% of their time for the study participants. Therefore, the objective was to have 40 active participants in the study. Since a drop-out rate of 20% is common in telephone interventions, the aim was to recruit 50 participants."

7b) When applicable, explanation of any interim analyses and stopping guidelines

Does your paper address CONSORT subitem 7b? *
No interim analyses were made.

8a) Method used to generate the random allocation sequence

NPT: When applicable, how care providers were allocated to each trial group

Does your paper address CONSORT subitem 8a? *
Yes "The eligible participants were randomly allocated either to a research (technology-assisted telephone intervention) or control (traditional telephone intervention) group in a 1:1 ratio, using stratified block randomization. Group allocation was stratified by socio-economic status and having minors as family members, since these factors were anticipated to influence the mental well-being and adherence outcomes of the study due to challenges in meeting the demands of work and family responsibilities "

8b) Type of randomisation; details of any restriction (such as blocking and block size)

Does your paper address CONSORT subitem 8b? *
Yes. "The eligible participants were randomly allocated either to a research (technology-assisted telephone intervention) or control (traditional telephone intervention) group in a 1:1 ratio, using stratified block randomization."

9) Mechanism used to implement the random allocation sequence (such as sequentially numbered containers), describing any steps taken to conceal the sequence until interventions were assigned

Does your paper address CONSORT subitem 9? *
Yes. "The participants were randomized simultaneously to the two groups via Microsoft Excel (2010) using its random number generator."

10) Who generated the random allocation sequence, who enrolled participants, and who assigned participants to interventions

Does your paper address CONSORT subitem 10? *

Yes. " The randomization was conducted by a researcher, who was not involved in the study as an investigator."

11a) If done, who was blinded after assignment to interventions (for example, participants, care providers, those assessing outcomes) and how

NPT: Whether or not administering co-interventions were blinded to group assignment

11a-i) Specify who was blinded, and who wasn’t

Specify who was blinded, and who wasn’t. Usually, in web-based trials it is not possible to blind the participants [1, 3] (this should be clearly acknowledged), but it may be possible to blind outcome assessors, those doing data analysis or those administering co-interventions (if any).
subitem not at all important 1 2 3 4 5 essential

Does your paper address subitem 11a-i? *

No. It was not possible to blind the participants and coaches were not blinded. Also, the researchers performing the data analyses were not blinded.

11a-ii) Discuss e.g., whether participants knew which intervention was the “intervention of interest” and which one was the “comparator”

Informed consent procedures (4a-ii) can create biases and certain expectations - discuss e.g., whether participants knew which intervention was the “intervention of interest” and which one was the “comparator”.
subitem not at all important 1 2 3 4 5 essential

Does your paper address subitem 11a-ii?

Yes. Information of the two interventions and the research intervention of interest were provided in the informed consent. "Registered employees received informed consents by regular mail, where information was provided regarding the two study groups, intervention, data collection, data processing, data privacy, research partners and contact details."

11b) If relevant, description of the similarity of interventions

(this item is usually not relevant for ehealth trials as it refers to similarity of a placebo or sham intervention to a active medication/intervention)

Does your paper address CONSORT subitem 11b? *

Yes. "The main difference between the interventions was the number of telephone calls and the use of technology in the coaching. The research group had five coaching calls during the intensive phase and one in the end. The control group had five coaching calls in the intensive phase and tree in the maintenance phase."

12a) Statistical methods used to compare groups for primary and secondary outcomes

NPT: When applicable, details of whether and how the clustering by care providers or centers was addressed

Does your paper address CONSORT subitem 12a? *

Yes. "For the primary trial outcome, mental well-being (WorkOptimum index), Mann-Whitney U tests were conducted … to determine the statistical significance of the between-group differences"… "Similar between-group analyses were performed for the time use of coaches, participants' adherence (frequency and diligence) to and satisfaction with coaching. " …" In addition, the statistical significance of the within-group changes in mental well-being from baseline (month 0) to the end of the intensive (month 4) and the maintenance phases (month 9) were determined with the Sign test."… "The Vargha-Delaney A measure of stochastic superiority is reported as an indicator for the effect size. For the between-group analyses, also the 95% confidence intervals (CI) of the effect sizes are reported. ."

12a-i) Imputation techniques to deal with attrition / missing values
Imputation techniques to deal with attrition / missing values: Not all participants will use the intervention/comparator as intended and attrition is typically high in ehealth trials. Specify how participants who did not use the application or dropped out from the trial were treated in the statistical analysis (a complete case analysis is strongly discouraged, and simple imputation techniques such as LOCF may also be problematic [4]).
subitem not at all important 1 2 3 4 5 essential

Does your paper address subitem 12a-i? *
Yes. " For the different outcomes, all the participants with relevant data available were included in the analyses (available-cases analysis). "

12b) Methods for additional analyses, such as subgroup analyses and adjusted analyses

Does your paper address CONSORT subitem 12b? *
No, there were no subgroup analyses performed.

X26) REB/IRB Approval and Ethical Considerations [recommended as subheading under "Methods"] (not a CONSORT item)

X26-i) Comment on ethics committee approval
subitem not at all important 1 2 3 4 5 essential

Does your paper address subitem X26-i?
Yes. "The study was evaluated and approved by the Ethics Committee of Human Sciences at the University of Oulu."

x26-ii) Outline informed consent procedures
Outline informed consent procedures e.g., if consent was obtained offline or online (how? Checkbox, etc.?), and what information was provided (see 4a-ii). See [6] for some items to be included in informed consent documents.
subitem not at all important 1 2 3 4 5 essential

Does your paper address subitem X26-ii?
Yes. "Informed consent was obtained from the interested individuals by regular mail before administering the electronic eligibility survey via e-mail."

X26-iii) Safety and security procedures
Safety and security procedures, incl. privacy considerations, and any steps taken to reduce the likelihood or detection of harm (e.g., education and training, availability of a hotline)
subitem not at all important 1 2 3 4 5 essential

Does your paper address subitem X26-iii?
No. Research data was anonymised, data was stored in secure digital environment, located in EU. Only researchers in project group had access to the data. The existing services of Luona (former Mawell Care Ltd.), Movendos and Firstbeat have their privacy policies for the used services: www.luona.fi/wp-content/uploads/2020/11/Tietosuojaseloste_Markkinointi_nettisivuille-12.11.20.pdf , materials.movendos.com/mCoach/MovendosPrivacyPolicy.pdf , www.firstbeat.com/en/privacy/firstbeat-lifestyle-assessment-privacy-policy/

RESULTS

13a) For each group, the numbers of participants who were randomly assigned, received intended treatment, and were analysed for the primary outcome

NPT: The number of care providers or centers performing the intervention in each group and the number of patients treated by each care provider in each center
Does your paper address CONSORT subitem 13a? *
Yes. "In total, 131 volunteers registered for the study, of which 56 met the inclusion criteria and were randomized equally to research and control groups."… "50 participants were chosen to be enrolled in the study based on the order of registration."… "At the beginning of the coaching program, one participant in the research group was no longer eligible for the study due toa change in their employment status and thus was omitted from the statistical analyses. " in Figure 3, primary analyses are marked for the research group n=21, control, n=19.

13b) For each group, losses and exclusions after randomisation, together with reasons

Does your paper address CONSORT subitem 13b? (NOTE: Preferably, this is shown in a CONSORT flow diagram) *
Yes. "At the beginning of the coaching program, one participant in the research group was no longer eligible for the study due to a change in their employment status and thus was omitted from the statistical analyses."

13b-i) Attrition diagram
Strongly recommended: An attrition diagram (e.g., proportion of participants still logging in or using the intervention/comparator in each group plotted over time, similar to a survival curve) or other figures or tables demonstrating usage/dose/engagement.
subitem not at all important 1 2 3 4 5 essential

Does your paper address subitem 13b-i?
No. The participant flow diagram (Figure 3) shows the participant adherence flow for the assessments and analyses for mental well-being analyses. The Movendos use was not tracked.

14a) Dates defining the periods of recruitment and follow-up

Does your paper address CONSORT subitem 14a? *
Yes. "The trial registration opened in November 2014, the trial started in February 2015 and ended in October 2015."

14a-i) Indicate if critical "secular events" fell into the study period
Indicate if critical "secular events" fell into the study period, e.g., significant changes in Internet resources available or "changes in computer hardware or Internet delivery resources"

subitem not at all important 1 2 3 4 5 essential

Does your paper address subitem 14a-i?
No. No significant changes took place.

14b) Why the trial ended or was stopped (early)

Does your paper address CONSORT subitem 14b? *
No, not applicable since the trial rolled out as planned.

15) A table showing baseline demographic and clinical characteristics for each group

NPT: When applicable, a description of care providers (case volume, qualification, expertise, etc.) and centers (volume) in each group
Does your paper address CONSORT subitem 15? *
Yes. "The baseline characteristics of the participants are presented in Table 4."

15-i) Report demographics associated with digital divide issues
In ehealth trials it is particularly important to report demographics associated with digital divide issues, such as **age, education, gender, social-economic status, computer/Internet/ehealth literacy of the participants**, if known.
subitem not at all important 1 2 3 4 5 essential

Does your paper address subitem 15-i? *
Yes. "The baseline characteristics of the participants are presented in Table 4. Most of the participants (95.92%, 47/49) were female, and the two males were allocated to the control group. More than half (57.14%, 28/49) of the participants were aged 46 to 60 years (mean age 46.26 years, SD 9.74). Most had at least the bachelor's degree (83.67%, 41/49)."

16) For each group, number of participants (denominator) included in each analysis and whether the analysis was by original assigned groups

16-i) Report multiple "denominators" and provide definitions
Report multiple "denominators" and provide definitions: Report N's (and effect sizes) "across a range of study participation [and use] thresholds" [1], e.g., N exposed, N consented, N used more than x times, N used more than y weeks, N participants "used" the intervention/comparator at specific pre-defined time points of interest (in absolute and relative numbers per group). Always clearly define "use" of the intervention.
subitem not at all important 1 2 3 4 5 essential

Does your paper address subitem 16-i? *
Yes, numbers of analyzed persons, statistical significance, Effect sizes etc. have been reported in Table 4 and 5.

16-ii) Primary analysis should be intent-to-treat
Primary analysis should be intent-to-treat, secondary analyses could include comparing only "users", with the appropriate caveats that this is no longer a randomized sample (see 18-i).
subitem not at all important 1 2 3 4 5 essential

Does your paper address subitem 16-ii?

Yes. "Figure 3 summarizes the participant flow from registration to available-case analysis for mental well-being as the primary outcome, and the attrition numbers for the intensive and the maintenance phases together with reasons for withdrawal."

17a) For each primary and secondary outcome, results for each group, and the estimated effect size and its precision (such as 95% confidence interval)

Does your paper address CONSORT subitem 17a? *

Yes. In Table 5 and 6, these are presented.

17a-i) Presentation of process outcomes such as metrics of use and intensity of use

In addition to primary/secondary (clinical) outcomes, the presentation of process outcomes such as **metrics of use and intensity of use** (dose, exposure) and their operational definitions is critical. This does not only refer to metrics of **attrition** (13-b) (often a binary variable), but also to more continuous exposure metrics such as "**average session length**". These must be accompanied by a technical description how a metric like a "session" is defined (e.g., timeout after idle time) [1] (report under item 6a).

subitem not at all important 1 2 3 4 5 essential

Does your paper address subitem 17a-i?

Yes. "The total time use of coaches … was not statistically significantly different between the two groups (366.0min vs 343.0min, $\hat{A}$=.60, 95% CI .33-.85, P =.48) (Table 5). However, the mean preparation time per coaching call was considerably higher for the research group ($\hat{A}$ = .90, 95% CI .75-1.0, P=.001). The mean duration per call did not differ between the groups ($\hat{A}$=.59, 95% CI .37-.80, P=.40). Regarding the personal coaching messages, the coaches sent altogether 60 and 102 personal coaching messages to the research group participants during the intensive and the maintenance phases, respectively."

17b) For binary outcomes, presentation of both absolute and relative effect sizes is recommended

**Does your paper address CONSORT subitem 17b? ***Kysymys on pakollinen.

No, this is not applicable for our study since we don't have binary outcomes.

18) Results of any other analyses performed, including subgroup analyses and adjusted analyses, distinguishing pre-specified from exploratory

Does your paper address CONSORT subitem 18? *

Not applicable, no subgroup analyses were made.

18-i) Subgroup analysis of comparing only users

A subgroup analysis of comparing only users is not uncommon in ehealth trials, but if done, it must be stressed that this is a self-selected sample and no longer an unbiased sample from a randomized trial (see 16-iii).

subitem not at all important 1 2 3 4 5 essential

Does your paper address subitem 18-i?

No. Not applicable since no subgroup analyses were made.

19) All important harms or unintended effects in each group

(for specific guidance see CONSORT for harms)

Does your paper address CONSORT subitem 19? *
No. Not applicable since no critical harms or problems rose.

19-i) Include privacy breaches, technical problems
Include privacy breaches, technical problems. This does not only include physical “harm” to participants, but also incidents such as perceived or real privacy breaches [1], technical problems, and other unexpected/unintended incidents. “Unintended effects” also includes unintended positive effects [2].
subitem not at all important 1 2 3 4 5 essential

Does your paper address subitem 19-i?
No, since nothing critical came up.

19-ii) Include qualitative feedback from participants or observations from staff/researchers
Include qualitative feedback from participants or observations from staff/researchers, if available, on strengths and shortcomings of the application, especially if they point to unintended/unexpected effects or uses. This includes (if available) reasons for why people did or did not use the application as intended by the developers.
subitem not at all important 1 2 3 4 5 essential

Does your paper address subitem 19-ii?
No, since we concentrated more on the quantitative data. Adding qualitative data would require more data analyses.

DISCUSSION

22) Interpretation consistent with results, balancing benefits and harms, and considering other relevant evidence

NPT: In addition, take into account the choice of the comparator, lack of or partial blinding, and unequal expertise of care providers or centers in each group
22-i) Restate study questions and summarize the answers suggested by the data, starting with primary outcomes and process outcomes (use)
Restate study questions and summarize the answers suggested by the data, starting with primary outcomes and process outcomes (use).
subitem not at all important 1 2 3 4 5 essential

Does your paper address subitem 22-i? *
Kysymys on pakollinen.
Lisäsin tähän tekstiä Discussion alkuun:
Yes. ” The aim of this study was to investigate whether the technology-assisted telephone intervention (C2) is feasible for increasing well-being or decreasing the time use of coaches while maintaining adherence and satisfaction compared with the traditional telephone intervention (C1) in an occupational healthcare setting..”

22-ii) Highlight unanswered new questions, suggest future research
Highlight unanswered new questions, suggest future research.
subitem not at all important 1 2 3 4 5 essential

Does your paper address subitem 22-ii?

Yes. “For a comprehensive cost-effectiveness study, the costs should be studied more widely from both the care provider and societal aspects. Also, the technology itself will bring costs which have to be considered.” “It should be also explored which coaching activities could be further automized to maintain the effects but decrease the time use of coaches. . " “There is need for reliable objective measures for the time use of coaches.”

20) Trial limitations, addressing sources of potential bias, imprecision, and, if relevant, multiplicity of analyses

20-i) Typical limitations in ehealth trials

Typical limitations in ehealth trials: Participants in ehealth trials are rarely blinded. Ehealth trials often look at a multiplicity of outcomes, increasing risk for a Type I error. Discuss biases due to non-use of the intervention/usability issues, biases through informed consent procedures, unexpected events.
subitem not at all important 1 2 3 4 5 essential

Does your paper address subitem 20-i? *

Yes. “One limitation is the narrow approximation of costs by the time use of coaches.” " Since the study was not blinded.”…” Because the utilized technology components and the technology-assisted coaching process was new to the coaches, the study might not provide realistic results on the time use of the coaches.”…” Additionally, there is a risk of error when self-reporting instead of objective measurements. In addition, data collection for the adherence required improvement during the pilot because measurement scales for evaluating adherence were ambiguous during the intensive phase.”…” The sample size was small, which makes the results only preliminary”… "

21) Generalisability (external validity, applicability) of the trial findings

NPT: External validity of the trial findings according to the intervention, comparators, patients, and care providers or centers involved in the trial

21-i) Generalizability to other populations

Generalizability to other populations: In particular, discuss generalizability to a general Internet population, outside of a RCT setting, and general patient population, including applicability of the study results for other organizations
subitem not at all important 1 2 3 4 5 essential

Does your paper address subitem 21-i?

Yes.
“The results were obtained in the coaching environment where phone calls were the primary means of coaching and may not be generalized to other forms of coaching. Additionally, coaching can be implemented in various ways, which makes the proper comparisons of different interventions or studies hard. Individual situations and health statuses vary quite a lot between people and here the participants were having moderate baseline well-being.

21-ii) Discuss if there were elements in the RCT that would be different in a routine application setting

Discuss if there were elements in the RCT that would be different in a routine application setting (e.g., prompts/reminders, more human involvement, training sessions or other co-interventions) and what impact the omission of these elements could have on use, adoption, or outcomes if the intervention is applied outside of a RCT setting.
subitem not at all important 1 2 3 4 5 essential

Does your paper address subitem 21-ii?
Yes. "Iit is expected that the process has the possibility to save coaches' time once the technology and process has been honed and become more of a routine."

OTHER INFORMATION

23) Registration number and name of trial registry

Does your paper address CONSORT subitem 23? *
Yes. "ClinicalTrials.gov NCT02445950" "The RCT was registered with the ClinicalTrials.gov (NCT02445950)."

24) Where the full trial protocol can be accessed, if available

Does your paper address CONSORT subitem 24? *
No. The study protocol has not been published in the form of scientific publication. Some details can be found though in the clinical trials registry.

25) Sources of funding and other support (such as supply of drugs), role of funders

Does your paper address CONSORT subitem 25? *
Yes. "This study was supported by the ARTEMIS-IA (Advanced Research & Technology for EMbedded Intelligent Systems Industry Association) and TEKES (currently Business Finland) under grant 332885 (WITH-ME)."

X27) Conflicts of Interest (not a CONSORT item)

X27-i) State the relation of the study team towards the system being evaluated
In addition to the usual declaration of interests (financial or otherwise), also state the relation of the study team towards the system being evaluated, i.e., state if the authors/evaluators are distinct from or identical with the developers/sponsors of the intervention.
subitem not at all important 1 2 3 4 5 essential

Does your paper address subitem X27-i?
Yes. "Ulla-Maija Junno is working at Luona Hoiva Ltd. (previously Mawell Care Ltd.), which provided the coaching for the intervention and Hannu Nieminen is working at Movendos Ltd., which provided the web coaching platform for the intervention. However, they have not been involved in the statistical analyses of the data."

About the CONSORT EHEALTH checklist

As a result of using this checklist, did you make changes in your manuscript? *

- **yes, major changes**
- yes, minor changes
- no

What were the most important changes you made as a result of using this checklist?

How much time did you spend on going through the checklist INCLUDING making changes in your manuscript *
The checklist was already used in the writing process and also updated now after review. Many frustrated extra hours were spend with this.

As a result of using this checklist, do you think your manuscript has improved? *

- **yes**
- no
- Muu:

Would you like to become involved in the CONSORT EHEALTH group?
This would involve for example becoming involved in participating in a workshop and writing an "Explanation and Elaboration" document

- yes
- **no**
- Muu:

Any other comments or questions on CONSORT EHEALTH
Filling this questionnaire is frustrating. Filling the questionnaire is taking too long (over 80 fields to fill + the multiple choices) and I quitted using the Google form in the end due to submission problems and I ended up working with Word document in the end.

STOP - Save this form as PDF before you click submit
To generate a record that you filled in this form, we recommend to generate a PDF of this page (on a Mac, simply select "print" and then select "print as PDF") before you submit it.

When you submit your (revised) paper to JMIR, please upload the PDF as supplementary file.

Don't worry if some text in the textboxes is cut off, as we still have the complete information in our database. Thank you!
Final step: Click submit !
Click submit so we have your answers in our database!
